# Identification of predictors of drug sensitivity using patient-derived models of esophageal squamous cell carcinoma

**DOI:** 10.1038/s41467-019-12846-7

**Published:** 2019-11-07

**Authors:** Dan Su, Dadong Zhang, Jiaoyue Jin, Lisha Ying, Miao Han, Kaiyan Chen, Bin Li, Junzhou Wu, Zhenghua Xie, Fanrong Zhang, Yihui Lin, Guoping Cheng, Jing-Yu Li, Minran Huang, Jinchao Wang, Kailai Wang, Jianjun Zhang, Fugen Li, Lei Xiong, Andrew Futreal, Weimin Mao

**Affiliations:** 1 Institute of Cancer and Basic Medicine (ICBM), Chinese Academy of Sciences, Hangzhou, Zhejiang China; 2 Department of Pathology, Cancer Hospital of the University of Chinese Academy of Sciences, Hangzhou, Zhejiang China; 3 Zhejiang Cancer Hospital, Hangzhou, Zhejiang China; 4 Research and Development Institute of Precision Medicine, 3D Medicines Inc., Shanghai, China; 5 Cancer Hospital of the University of Chinese Academy of Sciences, Hangzhou, Zhejiang China; 6 Department of Chemotherapy, Cancer Hospital of the University of Chinese Academy of Sciences, Hangzhou, Zhejiang China; 7 Department of Breast Surgery, Cancer Hospital of the University of Chinese Academy of Sciences, Hangzhou, Zhejiang China; 8 Department of Genomic Medicine, University of Texas MD Anderson Cancer Center, Houston, Texas USA; 9 Department of Thoracic/Head and Neck Medical Oncology, University of Texas MD Anderson Cancer Center, Houston, Texas USA; 10 Honorary Faculty, Wellcome Trust Sanger Institute, Hinxton, UK; 11 Department of Thoracic Surgery, Cancer Hospital of the University of Chinese Academy of Sciences, Hangzhou, Zhejiang China

**Keywords:** Cancer models, Cancer genomics, Oesophageal cancer, Predictive markers, Oesophageal cancer

## Abstract

Previous studies from the Cancer Cell Line Encyclopedia (CCLE) project have adopted commercial pan-cancer cell line models to identify drug sensitivity biomarkers. However, drug sensitivity biomarkers in esophageal squamous cell carcinoma (ESCC) have not been widely explored. Here, eight patient-derived cell lines (PDCs) are successfully established from 123 patients with ESCC. The mutation profiling of PDCs can partially recapture the tumor tissue actionable mutations from 161 patients with ESCC. Based on these mutations and relative pathways in eight PDCs, 46 targeted drugs are selected for screening. Interestingly, some drug and biomarker relationships are established that were not discovered in the CCLE project. For example, *CDKN2A* or *CDKN2B* loss is significantly associated with the sensitivity of CDK4/6 inhibitors. Furthermore, both PDC xenografts and patient-derived xenografts confirm *CDKN2A/2B* loss as a biomarker predictive of CDK4/6 inhibitor sensitivity. Collectively, patient-derived models could predict targeted drug sensitivity associated with actionable mutations in ESCC.

## Abstract (web summary)

Predicting the drug response of patients with cancer is crucial for implementing targeted therapy. Here, Su et al. make patient-derived cell lines and perform targeted sequencing and RNA-seq to identify *CDKN2A/2B* loss as a predictor of response to CDK4/6 inhibitors in esophageal squamous cell carcinoma.

## Introduction

Systemic studies of chemical compounds and genomic alternation have been conducted thoroughly by screening a large set of compounds against hundreds of commercial cell lines^1–3^. Many known agents associated with driven gene mutations were re-confirmed such as gefitinib and the *EGFR* (epidermal growth factor receptor) mutation, and some relationships, which have not been reported before, were also discovered^1–3^. In the meantime, these relationships could be limited by multiple cell lines across pan caner types due to heterogeneity and the loss of originality of commercial cell lines. For example, Garnett et al.^2^ adopted a pan-cancer cell model to perform a systematic identification of genomic markers of drug sensitivity, which might be limited in the clinical study of a single type of cancer.

Commercial cell lines are limited and might lose their original tumor characteristics due to repeated passaging, which results in genetic variation and divergence from the original tumor^4^. Therefore, cell biology assays and xenograft mouse models based on commercial cell lines might not be informative enough^5^. Patient-derived cell lines (PDCs) with low passage could be good alternatives to commercially available cell lines because they are directly derived from fresh tumor tissues^6,7^, inheriting the complexity and genetic diversity of the original tumor^8,9^. As a valuable experimental material, the success rate of PDC establishment directly isolated from tumor tissue samples is low^10,11^. Our previous study demonstrated that PDC-based models have been applied to elucidate the sensitivity of cells to various therapeutic agents^12^. Additionally, patient-derived xenografts (PDXs) are created when patient-derived cancerous tissue without prior digestion or in vitro culture are implanted directly into an immunodeficient mouse and could be used as extensively annotated models for pre-clinical analysis of therapeutics^13–15^. Hence, this study established an approach using patient-derived cells and models to explore the biomarkers of drug sensitivity and validate the results in esophageal squamous cell carcinoma (ESCC).

ESCC is the third most common cancer type in China^16^. Despite recent improvements in multimodal therapy, the 5-year overall survival (OS) rate of ESCC patients is only 25–40%^17^. There are currently no effective targeted drugs approved for ESCC. Recent genomic studies on ESCC have revealed frequently mutated cancer genes^18–24^, as well as recurrent somatic copy number variations (CNVs) at 11q13.2-q13.4 and 9p21.3^23^. Although uncovering of the genomic landscape and functional study of these mutant cancer genes have deepened our understanding of the mechanism of ESCC occurrence and development, further exploration and validation of these genes as potential therapeutic biomarkers of drug sensitivity for ESCC are largely lacking. In particular, an effective method of biomarker exploration and validation is absent.

A number of drugs for screening were limited in the actionable mutations and related pathways^25–28^. In this study, only targeted deep sequencing focused on tumor-related genes was an effective approach to identifying genomic variants associated with cancer tumorigenesis in a large cohort of ESCC and their PDCs. One aim is to understand the landscape of tumor-related genomic alternations in ESCC and another is to discover the actionable mutations for drug screening in PDCs to establish the relationship between drug and mutation. The selected biomarker and corresponding drug was further validated in vitro and in vivo. This study demonstrated that deep sequencing combined with patient-derived models can identify potential biomarkers of targeted drug sensitivity in ESCC.

## Results

### Cancer gene mutations in ESCC

To investigate the somatic cancer gene mutation landscape of ESCCs, we performed next-generation sequencing (NGS) of 161 tumor samples paired with a matching peripheral blood sample as a normal control, on a panel targeting 365 tumor-associated genes (Table 1, Supplementary Data 1 and 2). The mean sequencing depth of the tumors and of the matched blood DNA samples were 394× and 431×, respectively (Supplementary Data 3). A total of 2121 unique somatic mutations (Supplementary Data 4) was discovered with 57% missense mutations, 30% synonymous mutations, 7% stop-gain mutations, and 3% splice sites (Supplementary Fig. 1a), which are compatible with previous studies^18–20^. The most recurrent base substitution is the C > T transition (Supplementary Fig. 1b).

**Table 1:** Clinicopathological features of 161 patients for profiling cancer gene mutations

Factors	No. of patients	%
Gender		
Male	140	87.0
Female	21	13.0
Age (years)		
≤65	128	79.5
>65	33	20.5
Mean, median (range)	60.2, 61.0 (43–79)	
Body mass index (kg/m^2^)		
<18.5	30	18.6
18.5–25	119	73.9
>25	11	6.8
Missing	1	0.6
Mean, median (range)	21.0, 20.8 (15.4–29.3)	
Smoking history		
No	36	22.4
Yes	124	77.0
Missing	1	0.6
Alcohol intake		
No	43	26.7
Yes	117	72.7
Missing	1	0.6
Family history		
No	112	69.6
Yes	48	29.8
Missing	1	0.6
Tumor location		
Upper	13	8.1
Middle	96	59.6
Lower	52	32.3
Grade		
Well	2	1.2
Moderate	118	73.3
Poor	37	23.0
Missing	4	2.5
Clinical stage		
I	1	0.6
II	4	2.5
IIIa	91	56.5
IIIb	47	29.2
IIIc	18	11.2

**Fig. 1:**
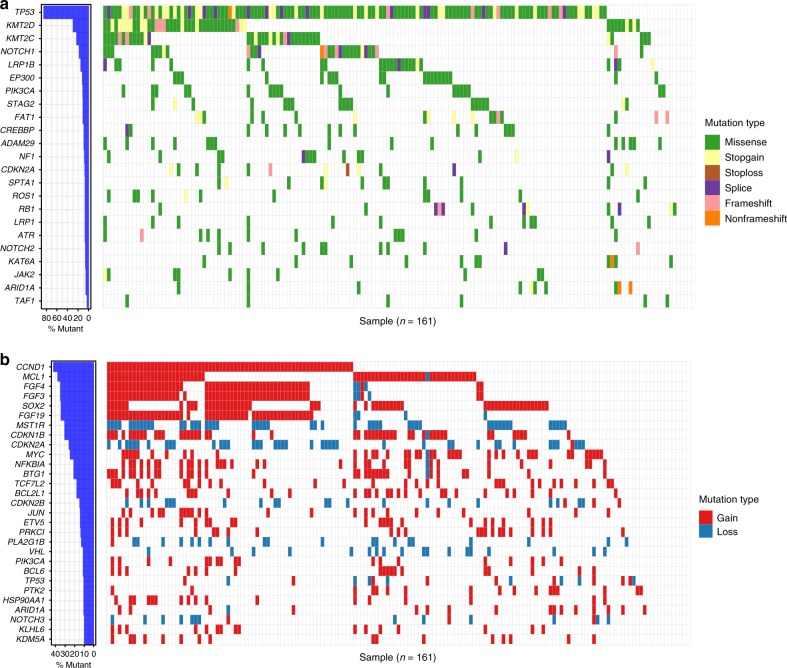
Top recurrent genes harbored somatic variants and somatic CNVs. **a** Left panel, bar plot shows the proportion of 161 ESCC samples with somatic mutations in the specific genes. Right panel, occurrence of the top 23 ranked somatically mutated genes identified by the cancer panel. Mutation subtypes (Missense, Stopgain, Stoploss, Splice, Frameshift, and Non-frameshift) are denoted by color. **b** Heatmap of the top recurrent genes associated with the top recurrent somatic CNVs in the 161 ESCC samples. The genes with recurrence of more than 10% are shown here. Mutation subtypes (Gain and Loss) are denoted by color

The most frequently mutated genes in the cohort of 161 samples (Fig. 1a) were also frequently observed in previous studies of ESCC^18–21,23^. These included *TP53*, *KMT2D* (*MLL2*), *KMT2C* (*MLL3*), *NOTCH1*, *LRP1B*, *EP300*, *PIK3CA*, *FAT1*, *CREBBP*, *ADAM29*, *RB1*, *NOTCH2*, and others (Fig. 1a). Out of 161 ESCCs, 153 (95%) samples have at least one somatic CNV. Within these CNVs, eight CNVs have recurrence rates ≥30% (Fig. 1b and Supplementary Data 5). Among all genes with copy number alterations, *CCND1* (42%), *MCL1* (38%), *FGF4* (35%), *FGF3* (35%), *SOX2* (34%), *FGF19* (34%), and *CDKN1B* (30%) were frequently amplified, while *MST1R* (30%), *CDKN2A* (26%), and *CDKN2B* (13%) were recurrently deleted (Fig. 1b).

To investigate the associations of mutations with clinical outcomes, a survival analysis based on the genetic profile of the 161 ESCC patients was carried out. We found that *PIK3CA* amplification and *JUN* neutral were significantly associated with poorer disease-free survival (DFS, *p* = 0.036 and 0.043, log-rank test) and OS (OS, *p* = 0.023 and 0.018, log-rank test) (Supplementary Fig. 2a, b). ESCC patients with *H3F3A* amplification had a significantly shortened DFS (*p* = 0.003) and a trend of shortened OS (*p* = 0.160, log-rank test) in comparison to those harboring *H3F3A* neutral (Supplementary Fig. 2c). ESCC patients with *NOTCH2* mutations had a significantly shortened OS in comparison to those without *NOTCH2* mutations (*p* = 0.034, log-rank test, Supplementary Fig. 2d). In addition, we found that *ACVR2A* amplification was associated with poorer DFS (*p* = 0.038, log-rank test, Supplementary Fig. 2e), while *DAXX* amplification was associated with poorer OS (*p* = 0.050, log-rank test, Supplementary Fig. 2f).

### Study of the potential biomarkers of drug sensitivity

**Fig. 2:**
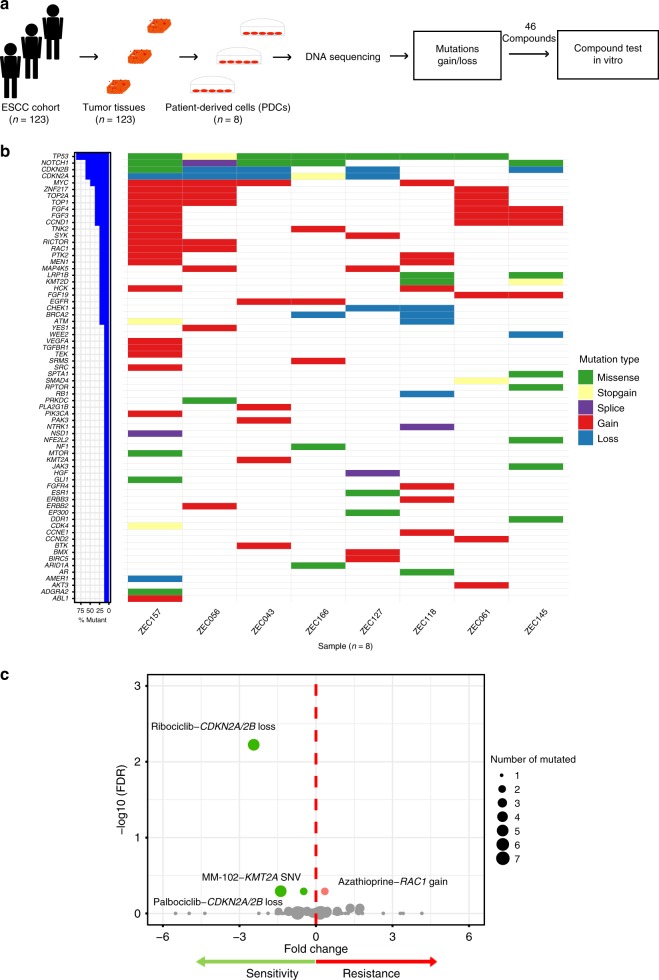
Exploration of the potential biomarkers of drug sensitivity in ESCC. **a** A schematic diagram exploring potential biomarkers of drug sensitivity in esophageal squamous cell carcinoma. Eight ESCC patient-derived cells (PDCs) were established from an independent cohort of 123 ESCC patients. DNA sequencing was used to detect the mutations and gain/loss in ESCC PDCs. We used the ESCC PDCs for integrated targeted deep sequencing and drug sensitivity evaluation systems to explore potential biomarkers of drug sensitivity. **b** The mutational landscape was studied in eight ESCC PDCs. **c** Each circle represents a single drug–gene interaction, and the size is proportional to the number of mutant cell lines screened (range 1–7). An unpaired *t* test was performed for each drug–gene mutation associations. The top four drug–gene mutation associations sorted by FDR were colored by green (sensitive) and red (resistant)

To further explore the potential biomarkers of drug sensitivity in ESCC, establishment and molecular characterization of PDCs were integrated with deep sequencing and drug sensitivity evaluation into an approach (Fig. 2a). We succeeded in deriving eight ESCC PDCs (ZEC043, ZEC056, ZEC061, ZEC118, ZEC127, ZEC145, ZEC157, and ZEC166) from the 123 ESCC patient tissues available, with a 6.5% success rate of establishing ESCC PDCs (Supplementary Data 6). Cell morphology, identification, and imaging results of karyotyping are distinct across PDCs (Supplementary Fig. 3a, b and Supplementary Data 7). Karyotype examination demonstrated that the number of chromosomes ranged from 34 to 85. Furthermore, comparison analyses of short tandem repeats (STRs) of these eight ESCC PDCs (Supplementary Data 8) with those from American Type Culture Collection, Deutsche Sammlung von Mikroorganismen und Zellkulturen, and other cell banks suggested that all eight ESCC PDCs are unique lines, devoid of cross-contamination with other known cancer cell lines. To confirm the origin of these PDCs, single-nucleotide polymorphisms (SNP) analysis demonstrated that SNPs in the PDCs were clustered with those in the corresponding tumor tissues (Supplementary Fig. 3c), confirming that the PDCs are derived from their corresponding tumor tissues without cross-sample contamination.

The somatic mutational landscape of these eight PDCs was profiled using targeted deep sequencing (Fig. 2b, Supplementary Data 9 and 10). These PDCs recaptured some mutational characteristics of the ESCC population. For example, the eight PDCs harbored the single-nucleotide variant (SNV)/indel overlapping genes found in the 161 ESCC patients (recurrence ≥5%), including *AR*, *ARID1A*, *CDKN2A*, *EP300*, *KMT2D*, *LRP1B*, *NF1*, *NOTCH1*, *SPTA1*, and *TP53* (Supplementary Fig. 4a). In addition, the CNV overlapping genes between the eight PDCs and the 161 ESCC patients (recurrence ≥10%) contained *MYC*, *FGF19*, *PTK2*, *CDKN2A*, *CDKN2B*, *CCND1*, *PLA2G1B*, *HSP90AA1*, *PIK3CA*, *FGF3*, and *FGF4* (Supplementary Fig. 4b). These results showed that ESCC PDCs could partially recapture the mutational characteristics of tumor tissues from 161 ESCC patients.

Based on the mutational landscape of the eight PDCs, a list of 46 compounds targeting mutated genes or their pathways was identified for a drug-sensitivity evaluation (Supplementary Data 11). The effect of 96 h of drug treatment on cell viability was used to derive the half-maximal inhibitory concentration (IC50) of drug sensitivity. In total, the IC50s of 46 compounds on ESCC PDCs ranged from 0.002 to 110.273 μM (Supplementary Data 12). To study the associations between the mutated genes and drug sensitivity across the ESCC PDCs, we used an analysis incorporating the ratio and significance of IC50s between mutated PDCs and non-mutated ones. This analysis revealed that there was an obvious gap of false discovery rate (FDR) level between top four drug–mutation associations sorted by FDR and other associations. The top four drug–mutation associations that have been preliminarily established were considered as the candidates (Fig. 2c and Supplementary Data 13). Interestingly, the two drug and biomarker relationships in four top candidates are associations involving *CDKN2A* or *CDKN2B* loss and CDK4/6 inhibitors (ribociclib and palbociclib). Moreover, there were five PDCs harboring *CDKN2A* or *CDKN2B* loss, three PDCs carrying no CNVs of *CDKN2A* and *CDKN2B*. Therefore, we focused on *CDKN2A* or *CDKN2B* loss as a potential biomarker of CDK4/6 inhibitor sensitivity.

### Validation of the biomarkers of drug sensitivity in vitro

For the CNVs of *CDKN2A* and *CDKN2B* in tumor tissue, we found that *CDKN2A* or *CDKN2B* loss was detected in 27% (44/161) of the ESCC samples (Fig. 1b). *CDKN2A* loss occurred in 41 ESCC formalin-fixed and paraffin-embedded (FFPE) samples (25%, 41/161), while *CDKN2B* loss was detected in 21 samples (13%, 21/161), with loss of both being found in 18 samples (11%, 18/161) (Supplementary Fig. 5a). It has been suggested that *CDKN2A* and *CDKN2B* tend to be lost together.

**Fig. 3:**
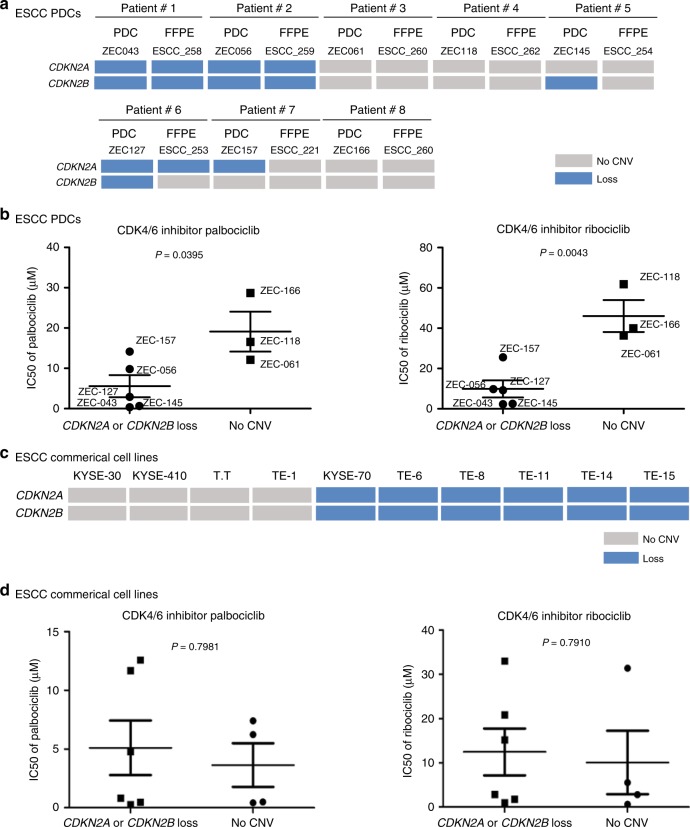
Validation of the biomarkers in PDCs and commercial cell lines. **a** The copy number variations (CNVs) of *CDKN2A* and *CDKN2B* were shown in eight ESCC PDCs and their corresponding tumor tissue. **b** Fifty percent growth inhibitory concentrations (IC50s) of palbociclib and ribociclib (LEE011) in eight ESCC PDCs with *CDKN2A* or *CDKN2B* loss or with no CNV of *CDKN2A* and *CDKN2B*. **c** The CNVs of *CDKN2A* and *CDKN2B* were illustrated in ten commercial cell lines. **d** IC50s of palbociclib and ribociclib in ten ESCC commercial cell lines with *CDKN2A* or *CDKN2B* loss or with no CNV of *CDKN2A* and *CDKN2B*. Error bars correspond to the standard deviation of IC50s. The comparisons between different groups of compound IC50s were performed using Student’s *t* test

To further validate potential CDK4/6 inhibitor sensitivity conferred by CNVs of *CDKN2A* and *CDKN2B*, two CDK4/6 inhibitors, palbociclib (PD-0332991) and ribociclib (LEE011), approved for clinical breast cancer use, were used to perform an analysis on the sensitivity of eight PDCs. We found that five out of eight PDCs (ZEC043, ZEC056, ZEC145, ZEC127, and ZEC157) harbored *CDKN2A* loss or *CDKN2B* loss (Fig. 3a). Due to intratumoral heterogeneity of tumor tissue and clonal enrichment of PDCs, some PDCs were not completely concordant with corresponding tumor tissue in detecting the CNVs of *CDKN2A* and *CDKN2B* (Fig. 3a). The average IC50s of palbociclib and ribociclib ranged from 0.361 to 28.669 μM and from 2.314 to 61.806 μM, respectively, in the eight ESCC PDCs (Supplementary Data 14). Interestingly, the IC50s of palbociclib (*p* = 0.0395, Student’s *t* test) in the *CDKN2A* or the *CDKN2B* loss group were significantly lower than in the wild-type group, while the IC50s of ribociclib (*p* = 0.0043, Student’s *t* test) in the *CDKN2A* or *CDKN2B* loss group were also significantly lower than in the wild-type group (Fig. 3b). In addition, there was no clinical characteristic of PDC corresponding patients associated with the sensitivities of palbociclib and ribociclib (Supplementary Data 15). Moreover, the other CDK4/6 selective-inhibitor abemaciclib was used to confirm the relationship with *CDKN2A/B* loss. The results showed that IC50s of abemaciclib (*p* = 0.0017, Student’s *t* test) in the *CDKN2A* or the *CDKN2B* loss group were significantly lower than in the wild-type group (Supplementary Fig. 5b and Supplementary Data 14), which was similar trend with the results from CDK4/6 inhibitors palbociclib and ribocicilib. On the other hand, mutation profiles of 10 ESCC commercial cell lines are available from Cancer Cell Line Encycloped (CCLE) project (Supplementary Data 16). Among 10 ESCC commercial cell lines, four ones with *CDKN2A*/*2B* wild-type and six ones with *CDKN2A*/*2B* loss were used to validate this biomarker (Fig. 3c). However, the results of drug-sensitivity evaluation showed that there was no significant difference in the IC50s of palbociclib (*p* = 0.7981, Student’s *t* test) and ribociclib (*p* = 0.7910, Student’s *t* test) between commercial cell lines with *CDKN2A*/*2B* wild-type and ones with *CDKN2A*/*2B* loss (Fig. 3d). The data suggest that the potential biomarker of CDK4/6 inhibitors could be validated in ESCC PDCs rather than commercial cell lines.

**Fig. 4:**
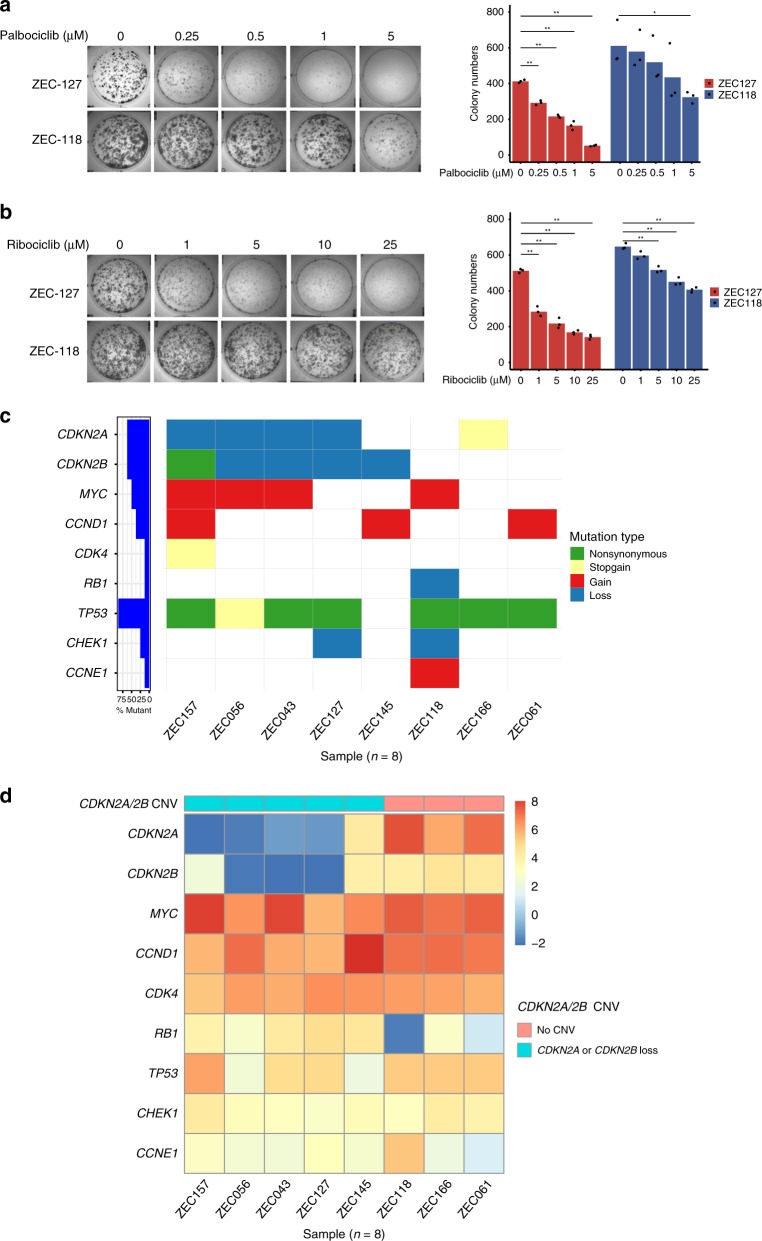
Validation of the biomarkers of drug sensitivity in vitro. **a**, **b** ZEC127 (*CDKN2A*/*2B* loss) and ZEC118 (*CDKN2A*/*2B* no CNV) were used to evaluate the sensitivity of CDK4/6 inhibitors (palbociclib and ribociclib) using colony formation assays. Three experiments were averaged, and error bars correspond to the standard deviation of colony numbers. The colony number differences between different dose groups of inhibitors were compared using a Student’s *t* test and *p < 0.01 and **p < 0.001. **c** Mutations of cell cycle checkpoint genes including *CDKN2A*, *CDKN2B*, *MYC*, *CCND1*, *CDK4*, *RB1*, *TP53*, *CHEK1*, and *CCNE1* were detected in eight ESCC PDCs using targeted deep sequencing. **d** *Z*-score analysis of the expression level of cell cycle checkpoint genes in the eight ESCC PDCs, which carried either *CDKN2A* or *CDKN2B* loss, or neither

For further validation of biomarkers of CDK4/6 inhibitors, ZEC127 (*CDKN2A*/*2B* loss) and ZEC118 (*CDKN2A*/*2B* wild-type) were used to evaluate the sensitivity of CDK4/6 inhibitors using a colony formation assay. The number of ZEC127 (*CDKN2A*/*2B* loss) colonies was very significantly reduced at the first (*p* < 0.001 and *p* < 0.001, Student’s *t* test), for the lowest dose of both palbociclib (0.25 μM) and ribociclib (1 μM) (Fig. 4a), while the number of ZEC118 (*CDKN2A*/*2B* wild-type) colonies maintained at the same level across a series dose, but reduced with higher doses of palbociclib (5 μM) and ribociclib (5 μM) (Fig. 4b). In addition, the result of ZEC166 (*CDKN2A*/*2B* wild-type) was a similar trend to the result of ZEC118 (Supplementary Fig. 5c). These results indicated that ESCC PDCs with *CDKN2A* or *CDKN2B* loss are sensitive to CDK4/6 inhibitors.

In order to investigate the possible effects of these differences on CDK4/6 inhibitor sensitivity caused by cell cycle checkpoint genes, somatic mutation, and whole transcriptome analyses of ESCC PDCs were performed using targeted deep sequencing and RNA-sequencing. We discovered that there are some mutations of cell cycle checkpoint genes, including *CDKN2A*, *CDKN2B*, *MYC*, *CCND1*, *CDK4*, *RB1*, *TP53*, *CHEK1*, and *CCNE1* in the eight PDCs (Fig. 4c). The messenger RNA (mRNA) expression of these genes in these PDCs is shown in Fig. 4d. Further analyses of the associations of gene somatic mutations and mRNA expression were performed. Interestingly, the expression levels of *TP53* non-synonymous mutations and *CHEK1* loss were significantly different between the mutated group and the non-mutated group (*p* = 0.0002 and *p* = 0.0399, Wilcoxon’s test) (Supplementary Data 17). Most of the cell cycle-related genes (*MYC*, *CCND1*, *CDK4*, *RB1*, and *CCNE1*) were not significantly different in the expression levels (Supplementary Data 17). Moreover, the mRNA expression of *CDKN2A* and *CDKN2B* in the five PDCs harboring *CDKN2A* or *CDKN2B* loss was significantly lower than in the three PDCs with no CNV of *CDKN2A* and *CDKN2B* (*p* = 0.0357 and *p* = 0.0357, Wilcoxon’s test, respectively, Supplementary Fig. 5d, e), indicating that *CDKN2A* or *CDKN2B* loss leads to their depleted expression on the transcriptional level. In addition, the expression of cell cycle checkpoint proteins and their phosphorylation after treating with CDK4/6 inhibitors was detected by means of western blotting. We found that there were reductions of RB, pRB, CDK2, and pCDK2 in PDCs-ZEC043, ZEC056, and ZEC127 (*CDKN2A*/*2B* loss) with increasing dosage of palbociclib (Supplementary Fig. 5f). These data support the idea that biomarkers of CDK4/6 inhibitors for ESCC patients could include a loss of *CDKN2A* or *CDKN2B* rather than mutations of other cell cycle checkpoint genes in vitro.

### Validation of the biomarkers of drug sensitivity in vivo

**Fig. 5:**
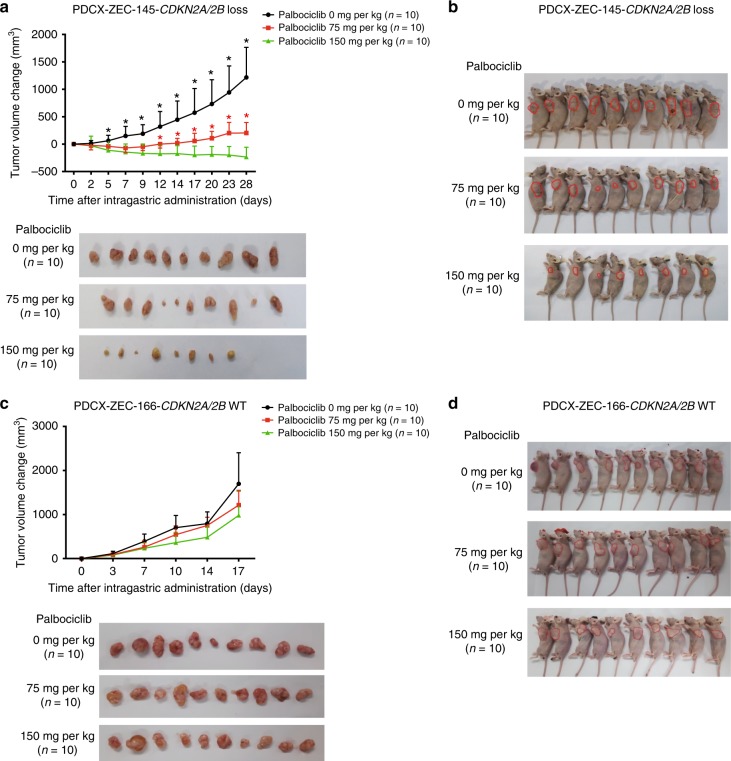
Validation of the biomarkers of drug sensitivity in vivo. **a**, **b** Change in tumor volume following treatment with palbociclib at three doses in ZEC145 PDCX *CDKN2A/2B* loss tumors (28 days) and in ZEC166 PDCX *CDKN2A/2B*-WT tumors (17 days) (mean ± s.e.m., *n* = 10). **c**, **d** General picture of ZEC145 PDCXs and ZEC166 PDCXs after intragastric administration of palbociclib (10 mice per dose). Error bars correspond to standard error of the mean of tumor volume. *p < 0.01, Student’s *t* test, palbociclib 0 mg/kg compared with palbociclib 75 mg/kg; *p < 0.01, Student’s *t* test, palbociclib 75 mg/kg compared with palbociclib 150 mg/kg

To confirm the results in vivo, ESCC PDC xenograft (PDCX) models were established. The two ESCC PDCs (ZEC145, with *CDKN2A*/*2B* loss, and ZEC166, harboring wild-type *CDKN2A*/*2B*) were both capable of generating tumor xenografts. Based on previous pre-clinical studies of palbociclib, dosages of 75 and 150 mg/kg were chosen for this study^29,30^. As expected, in PDCX-ZEC145 models with *CDKN2A* and *CDKN2B* loss, palbociclib given at a 75 mg/kg dose demonstrated a remarkable inhibition of tumor growth (Fig. 5a, b). When the dosage of palbociclib was increased to 150 mg/kg, more obvious regression of the subcutaneous tumors was found (Fig. 5a, b). However, in PDCX-ZEC166 models with wild-type *CDKN2A* and *CDKN2B*, the tumors still progressed in both palbociclib treatment groups (Fig. 5c, d). In addition, we observed the most apparent toxicity at the high dose of palbociclib (150 mg/kg) in both the *CDKN2A* and *CDKN2B* loss and the wild-type models (Supplementary Fig. 6a, b). In addition, after treatment with the CDK4/6 inhibitor palbocicilib of 75 mg/kg in vivo, the expression of Foxm1 was down-regulated in *CDKN2A/2B* loss xenografts (PDCX-ZEC145), but up-regulated in *CDKN2A/2B* wild-type xenografts (PDCX-ZEC166), while Smac levels increased in both xenografts. (Supplementary Fig. 6c). These data suggest that ESCC PDCX models with *CDKN2A* and *CDKN2B* loss are more sensitive to the CDK4/6 inhibitor palbociclib than the PDCX models with wild-type *CDKN2A* and *CDKN2B*.

### An ESCC PDX model confirms biomarkers of drug sensitivity

**Fig. 6:**
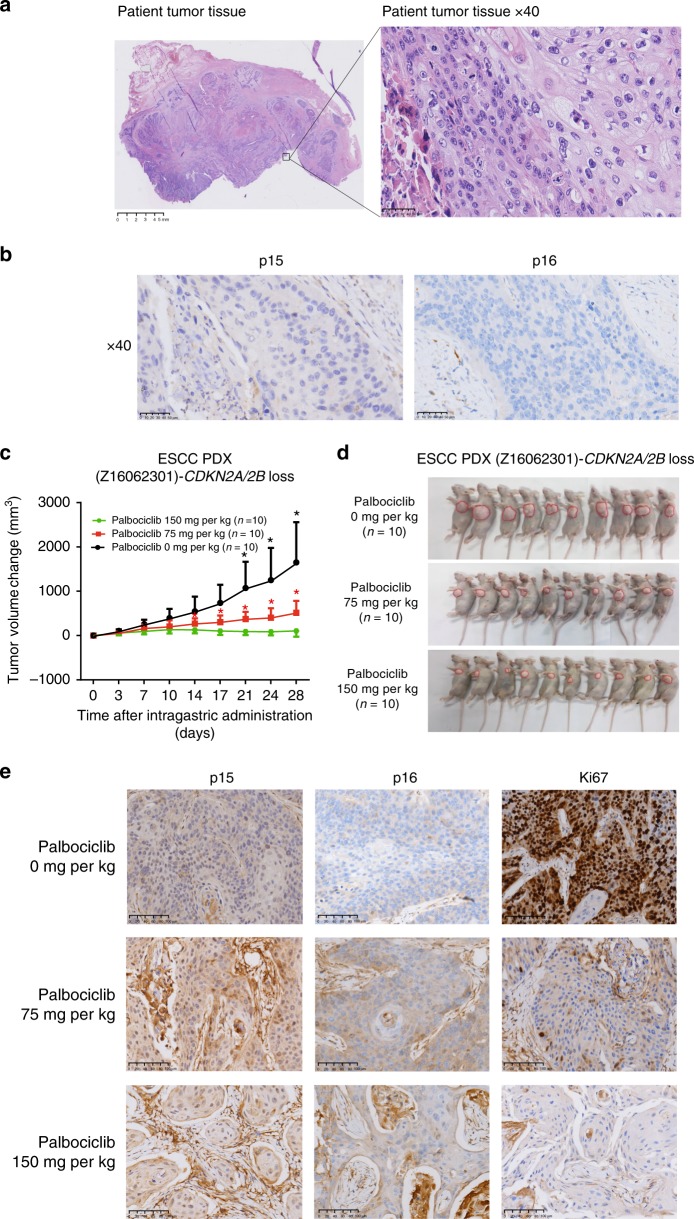
An ESCC PDX model to confirm the biomarkers of drug sensitivity. **a** Hematoxylin and eosin staining (HE) results of tumor tissue derived from an ESCC patient are shown. Magnification (right) is ×400. **b** The expression levels of p15 and p16 were detected by immunohistochemistry (IHC) staining in the FFPE tumor tissues of this ESCC patient. Magnification (right) is ×400. **c** Change in tumor volume following treatment with palbociclib in three doses in patient-derived xenografts (PDXs)-Z16062301 (28 days) (mean ± s.e.m., *n* = 10). Error bars correspond to standard error of the mean of tumor volume. *p < 0.01, Student’s *t* test, palbociclib 0 mg/kg compared with palbociclib 75 mg/kg; *p < 0.01, Student’s *t* test, palbociclib 75 mg/kg compared with palbociclib 150 mg/kg. **d** General picture of PDXs-Z16062301 after intragastric administration. **e** IHC staining of FFPE tumor tissue from PDXs treated with different dosages of palbociclib was used to detect the expression of p15, p16, and Ki67. The imaging results of IHC staining with the magnification of ×200 are shown

A male patient was diagnosed with ESCC in Zhejiang Cancer Hospital. He had an operation on 23 June 2016. The hematoxylin and eosin (HE) staining of his FFPE tumor tissue was shown in Fig. 6a. He had current smoking status without alcohol intake, and he had no family history of malignant tumors. His tumor was from the distal esophagus. Based on the seventh edition of the American Joint Committee on Cancer (AJCC) staging system for esophageal cancer^31^, he was stage IIIb (Supplementary Data 18). Mutational profiling was carried out by targeted deep sequencing (Supplementary Data 19) and showed that his tumor tissue harbored *CDKN2A* and *CDKN2B* loss. In addition, his tumor tissue was negative for both p15 (coded by *CDKN2B*) and p16 (coded by *CDKN2A*) by immunohistochemistry (IHC) staining (Fig. 6b). Unfortunately, he died before he could start taking CDK4/6 inhibitors. However, a PDX model (Z16062301) was successfully created by directly engrafting his surgically resected tumor tissues into immune-deficient mice. In addition, using targeted deep sequencing, we confirmed that the PDX-Z16062301 model had *CDKN2A* and *CDKN2B* loss.

As expected, in PDX-Z16062301 models, palbociclib treatment at a 75 mg/kg dose resulted in prominent inhibition of tumor growth with progression (Fig. 6c, d). When the dosage of palbociclib was increased to 150 mg/kg, more notable regression of the subcutaneous tumors was seen (Fig. 6c, d). We also observed the most apparent toxicity at this high dose of palbociclib (150 mg/kg) in both *CDKN2A* and *CDKN2B* loss and wild-type models (Supplementary Fig. 7a). Moreover, IHC staining of FFPE tumor tissue from PDXs treated with different dosages of palbociclib and potential quantification showed that p15 and p16 were almost stain free, and the number of cells that were Ki67 positive was reduced with increasing palbociclib dose (Fig. 6e and Supplementary Data 20). In addition, we found that expression levels of Foxm1 were decreased after treated with CDK4/6 inhibitor palbocicilib of 75 mg/kg in PDX-Z16062301 (Supplementary Fig. 7b). This mouse study of the ESCC PDX model confirms that *CDKN2A* and *CDKN2B* loss is a biomarker of CDK4/6 inhibitor sensitivity.

## Discussion

To identify the biomarkers of drug sensitivity in a CCLE project and accompanying reports, high-throughput drug screening was employed in commercialized cell lines from multiple types of cancer^1–3,32,33^. The biomarkers of drug sensitivity were systematically identified in that pan-cancer cell model. However, this approach is largely limited because there have been few pan-cancer biomarkers discovered so far such as *NTRK* fusion, MSI, and so on^34–39^. A majority of biomarkers are cancer-type-specific. For example, V600E BRAF melanoma but not colorectal cancers were sensitive to *BRAF*-targeting vemurafenib^40^. Amrita Basu et al.^3^ showed that *KRAS* mutations correlate significantly with sensitivity to navitoclax among colorectal cancer cell lines (CCLs), but not among all CCLs. In our study, we focused on a single cancer type, ESCC, using an approach composed of patient-derived models combined with targeted deep sequencing and drug-sensitivity evaluation systems.

We further analyzed the results of identifying the biomarkers of drug sensitivity by overlapping 14 compounds on 27 commercialized ESCC cell lines from the CCLE project and 8 ESCC PDCs from our platform. *P* values showing gene–drug sensitivity associations were adjusted using FDR correction for multiple testing. The results of the CCLE project showed that there was no gene–drug associations with drug sensitivity established in ESCC commercial cell lines (Supplementary Fig. 8a). Our data from the overlap of 14 compounds revealed an obvious FDR gap between the association of *CDKN2A* or *CDKN2B* loss with palbociclib for drug sensitivity and other gene–drug associations (Supplementary Fig. 8b). This association was further validated in ESCC patient-derived models in vitro and in vivo. These discrepant results suggest that commercialized cell line models have limitations in exploring the biomarkers of drug sensitivity, which could be overcome by PDC models. Despite the large cost, the PDC models are valuable and powerful in the identification of potential biomarkers of drug sensitivity.

Apart from association of *CDKN2A*/*2B* loss and CDK4/6 inhibitors, we have adopted this approach to discover other potential drug–genotype associations. We found that two *RAC1* gain PDCs were resistant to immunosuppressive agent azathioprine (also named thiopurine and 6-MP), which have been widely used in a variety of clinical conditions for decades, such as Crohn’s disease, rheumatic diseases, organ transplantation, and so on^41–44^. Razidlo et al.^45^ found that azathioprine was able to target pancreatic cancer metastasis through inhibition of Rac and Cdc42 signaling. In addition, it was reported that azathioprine induces resistance in hepatoblastoma cells to IGF-1, which leads to autophagy activation^46^. This study was the first to discover that azathioprine-*RAC1* gains associations in ESCC, which could help to expand the research of azathioprine in the anti-cancer field. Moreover, we still discovered that *KMT2D*-mutated PDCs were sensitive to the MLL1 (KMT2A) inhibitor MM-102 in ESCC, which was not found in the CCLE model. MLL1 is one of the six MLL family histone methyltransferases in mammals^47,48^. It was reported that cancer cell proliferation was dramatically lowered by pharmacological inhibition of the MLL1 methyltransferase complex^49^. However, the biomarker of MLL1 inhibitor sensitivity has not been reported. Our discovery of MM-102–*KMT2D* mutation associations may fill this gap and shed light on MLL1 inhibitor as a promising epigenetic treatment for ESCC patients.

The association of *CDKN2A* or *CDKN2B* loss with palbociclib for drug sensitivity was established in this study because we have a balance of five PDCs with *CDKN2A* or *CDKN2B* loss and three PDCs with *CDKN2A* or *CDKN2B* wild-type in the drug screening. Other associations were driven by dramatic responses in small numbers of outlier ESCC PDCs. For example, the only *ATM*-mutated PDC was exquisitely sensitive to the ATM inhibitor KU-55933, and the only *DDR1*-mutated PDC was sensitive to imatinib targeting v-Abl, c-Kit, and similar targets. Besides that, the only *NTRK1*-mutated PDC was resistant to MEK1/2 inhibitor trametinib, and the only *JAK3*-mutated PDC was resistant to JAK1/2 inhibitor Cyt387. These data confirm the need of large panels of ESCC PDCs with a balance of biomarker distribution to capture low-frequency drug–genotype associations. We believe that as PDCs are constantly established, which we are doing now, increasingly more gene–drug associations will be further discovered and validated in ESCC. This powerful approach has paved a way to discover and validate the biomarkers of drug sensitivity in ESCC as well as other cancers.

In this study, we established an approach composed of PDCs combined with targeted deep sequencing and a drug-sensitivity evaluation system in order to explore potential biomarkers of drug sensitivity in ESCC. To further validate these biomarkers of drug sensitivity, patient-derived models, targeted deep sequencing, RNA-sequencing, and drug-sensitivity evaluations were used in vitro and in vivo. Finally, a mouse trial consisting of PDXs was utilized to confirm our results. By this approach, *CDKN2A* or *CDKN2B* loss was discovered and identified to be a biomarker of CDK4/6 inhibitor sensitivity in ESCC. To our knowledge, this is the first time that researchers have integrated patient-derived models, targeted deep sequencing, RNA-sequencing, drug sensitivity evaluation systems, and mouse studies to identify potential biomarkers of drug sensitivity in relatively large ESCC cohorts. This approach, supported by its effective identification of drug–genotype associations, will lay a foundation for clinical testing of the biomarkers of targeted drug sensitivity.

## Methods

### Patient tumor tissue collection

One hundred and sixty-one FFPE tumor tissues and 123 fresh tumor tissues of 284 ESCC patients were obtained from the tissue bank of Zhejiang Cancer Hospital (Table 1 and Supplementary Data 6). Of these tissues, 161 with matched blood samples were used as controls to remove germline variants as a cohort (Table 1). The clinical information and follow-up for 10 years were collected for the 161 patients (Supplementary Data 1). All FFPE samples from this cohort were histologically examined by two senior pathologists independently and were confirmed to contain at least 20% tumor cells. In this cohort, 87.0% (140 patients) were male, 79.5% (128 patients) were ≤65 years old, 73.9% (119 patients) had body mass index from 18.5 to 25, 77.0% (124 patients) had current smoking status, 72.7% (117 patients) had current alcohol intake, 29.8% (48 patients) had a family history of ESCC, 59.6% (96 patients) had tumors which were from the middle esophagus, and 73.3% (118 patients) had tumors that were moderately differentiated. Based on the seventh edition of the AJCC staging system for esophageal cancer^31^, one patient (0.6%) was stage I, four patients (2.5%) were stage II, 91 patients (56.5%) were stage IIIa, 47 patients (29.2%) were stage IIIb, and 18 patients (11.2%) were stage IIIc. The median follow-up time for this cohort was 25.6 (range 0–88) months. The 3-year disease-free rate and 3-year survival rate for this cohort were 43.6% and 43.6%, respectively. The clinical information from an independent cohort of 123 ESCC patients, whose tumor tissues were used for constructing PDCs, is shown in Supplementary Data 6. All samples were collected with written informed consent, and the study was approved by the Institutional Review Board in Zhejiang Cancer Hospital.

### Deep sequencing and somatic mutation calling

The targeted panel consists of 365 cancer-related genes and 25 highly re-arranged genes in cancers (Supplementary Data 2). All sequencing assays were performed in 3DMed Medical Laboratory Co., Ltd (Shanghai), which successfully passed the tissue gene mutation testing capabilities Proficiency Testing on NGS solid tumors organized by American Association for pathology (CAP). DNA was isolated from FFPE slides containing at least 20% tumor cells^48^, PDCs, fresh tumor tissue samples from PDCX models and PDX models, and the blood samples from the corresponding patients. The library was prepared using IDTX gen hybridization buffer for capture and sequenced on an Illumina NextSeq 500.

FastQC software was used to evaluate the quality of sequencing data (http://www.bioinformatics.bbsrc.ac.uk/projects/fastqc/). Sequence reads from genomic DNA were mapped to human genome (hg19) reference using BWA-MEM^50^, and bam files were further processed by Picard (http://broadinstitute.github.io/picard/) to sort sequences and remove duplicated reads. Somatic SNVs were called by Mutect, and Indels (<50 bp) were identified by Pindel and VarScan^49–51^. Variant calls meeting the following criteria were advanced to further analysis: (1) the minimum coverage in tumors is 30; (2) the maximum mutation frequency in normal samples is ≤0.03; (3) the minimum mutation frequency difference between normal and tumor is ≥0.05; (4) the maximum strand bias is ≤0.9; and (5) the minimum support reads of mutation is ≥5. To remove more false positives, our in-house scripts were developed to filter out spurious SNVs near tandem repeat regions and indel regions. ANNOVAR software was utilized to facilitate variant annotation^52^ and variants with population frequencies >0.015 in the 1000 Genomes Project and in all subjects in the NHLBI-ESP Project with 6500 exomes were filtered out. Finally, we only considered variants annotated as non-synonymous, stop, gain and loss, synonymous, splice site, frameshift deletion and insertion, and non-frameshift deletion and insertion. For the variant calling, the blood samples from the corresponding patients were used as matched normal controls. For somatic CNVs, we applied a series of normalizations to sequencing coverage including matched normal sample, GC content, and segmentation. The coverage in the tumor was normalized to that in the matched normal, further normalization was done by nucleotide composition including GC content, and followed by segmentation and log ratio estimation, which were similar to ones described in BIC-Seq2^51^. Segment level CNV was defined as segments with log ratio >0.7 or <−0.7. The gene level CNV was defined as genes with >75% exons overlapping gain/loss segments. R package GenVisR was used to demonstrate somatic mutations spectrum and CNVs across ESCC cohorts^52^.

### Establishment and validation of primary cell lines

Tissues collected at the time of surgery and pleural effusions collected from palliative paracentesis were used for primary cell culture. Fresh tumor tissue was rinsed in phosphate-buffered saline (PBS) three times and minced into 0.5–1 mm^3^ pieces. For pleural effusion, the sediment was harvested and washed in PBS three times. Each sediment was then cultured in Dulbecco’s modified Eagle’s medium (Gibco) supplemented with 10% fetal bovine serum (Gibco), 1× non-essential amino acids (Gibco), 50 U/mL of penicillin (Gibco) and 50 μg/mL of streptomycin (Gibco), and maintained in a 37 °C, 5% CO_2_ incubator. After 5 to 7 days, cells were re-plated and designated as passage 0.

For STR genotyping, genomic DNA was extracted from each primary cell line using a QIAamp DNA Mini Kit (Qiagen Inc., Valencia, CA, USA). Nineteen STR loci (TH01, D12S391, D7S820, CSF1PO, FGA, D5S818, D2S1338, D21S11, D18S51, TPOX, vWA, D8S1179, D3S1358, D13S317, D6S1043, D16S539, Penta E, D19S433, and Penta D) and amelogenin were amplified by PCR and analyzed using an Applied Biosystems 3730xl DNA Analyzer (Applied Biosystems Inc., Foster City, CA, USA). For karyotyping, exponentially growing primary cells were exposed to colchicine (0.01 mg/mL) for 16 h and then to hypotonic treatment (0.075 mol/L KCl) for 20 min. After fixation in a methanol and acetic acid mixture (3:1 by volume), cell suspensions were dropped onto ice-cold slides. Slides were then treated in trypsin for 30–60 s and stained with Giemsa. Chromosomes from at least 20 metaphases per sample were analyzed under a microscope.

### In vitro cell viability assay with compounds

For cell viability studies, cells were plated in quadruplicate at a density of 4000 cells per well in 96-well plates in normal growth medium and allowed to adhere overnight. The compounds (CDK4/6 inhibitors) were then applied to cells at 10 different concentrations in a 3.3-fold dilution series. Cell viability was measured after 72 h of treatment using the CellTiter-Glo Luminescent Cell Viability Assay (Promega). The concentration of a drug resulting in 50% inhibition of cell viability (IC50) was calculated from a four-parameter curve analysis. Mean IC50 values and standard deviations of CDK4/6 inhibitors were calculated from four independent experiments for the in vitro validation.

In addition, ten ESCC commercial cell lines used in cell viability assay with compounds were purchased from three different cell banks, including European Collection of Authenticated Cell Cultures (ECACC), Japanese Collection of Research Bioresources (JCRB), and RIKEN BRC cell banks. The vendor of KYSE-30 (ECACC, cat. no. 94072011), KYSE-70 (ECACC, cat. no. 94072012), and KYSE-410 (ECACC, cat. no. 94072023) is Sigma-Aldrich (Shanghai) Trading Co., Ltd. The vendor of T.T (JCRB, cat. no. JCRB0262), TE-1 (RIKEN, cat. no. RCB1894), TE-6 (RIKEN, cat. no. RCB1950), TE-8 (RIKEN, cat. no. RCB2098), TE-11 (RIKEN, cat. no. RCB2100), TE-14 (RIKEN, cat. no. RCB2101), and TE-15 (RIKEN, cat. no. RCB1951) is 3DHTS Precision Medicine Institute.

### Colony formation assay

In total, 2000 cells per plate were seeded onto 6-well plates and treated with palbociclib (Med Chem Express Inc., USA; 0, 0.25, 0.5, 1, and 5 µM) for about 2 weeks. Then, the cells were washed with PBS twice, fixed with methanol for 10 min, and stained with 0.1% crystal violet for 15 min. The dishes were then washed with PBS at least three times. Photographs were captured, and cell clones containing >50 cells were counted.

### Drug sensitivity in vivo

ESCC PDCs (1 × 10^7^) were subcutaneously injected into the backs of male BALB/c nude mice, which were 4 weeks old with a weight of 17–20 g (Shanghai, China; license no., SCXK 2007-0005). Tumor size was monitored every 3 days, and the tumor growth curve and animal weight were recorded accordingly. Tumor volume (*V*) was calculated by the formula: *V* (mm^3^) = 1/2 (length × width^2^). After 7–14 days, when the tumor volume reached 1000 mm^3^, the mice were killed. Xenograft tumors were then collected and divided into 1–2 mm^3^ cubes, which were implanted subcutaneously into the left armpit of male BALB/c mice. After 2 weeks, 40 animals harboring 100–150 mm^3^ tumors were randomized into four groups receiving palbociclib treatment (Group 1, vehicle; Group 2, 75 mg/kg; Group 3, 150 mg/kg; administered by daily gavage). After 28 days or when the tumor volume had reached 1500 mm^3^, the experiments were ended, and both tumor and blood samples were reserved. Animal experiments were approved by the Institutional Animal Care and Use Committee of Zhejiang Chinese Medicine University (Hangzhou, Zhejiang, China).

### Western blot analysis

Cells were cultivated in 60-mm plates for 24 h, prior to treatment with palbociclib (Med Chem Express Inc., USA; 0, 0.5, 2.5, and 12.5 µM) for 48 h. The cells were then harvested and lysed in NP40 buffer with protease inhibitor cocktails. The concentration of total protein was measured using the Bradford colorimetric assay (Bio-Rad Laboratories, USA). Protein expression was detected using 8% sodium dodecyl sulfate-polyacrylamide gel electrophoresis. Subsequently, 20 µg total protein was transferred to polyvinylidene difluoride membranes (Millipore, USA), and the membranes were blocked for 120 min with freshly prepared 5% bovine serum albumin in Tris-buffered saline and Tween-20 (TBST). Following this, the membranes were incubated with antibodies at 4 °C overnight, washed three times with TBST, and incubated with goat anti-rabbit immunoglobulin G-horseradish peroxidase-conjugated antibodies (Google Biology Technology Inc., China). The following antibodies were used: pRb (Ser780) (diluted 1:1000; cat. no. 8180S; Cell Signaling Technology Inc., USA), pRb (Ser807/811) (diluted 1:1000; cat. no. 8516S; Cell Signaling Technology Inc., USA), Rb (diluted 1:800; cat. no. 9313S; Cell Signaling Technology Inc., USA), p-CDK2 (diluted 1:1000; cat. no. 2561S; Cell Signaling Technology Inc., USA), CDK2 (diluted 1:1000; cat. no. 2546S; Cell Signaling Technology Inc., USA), cyclin E1 (diluted 1:2000; cat. no. 11554-1-AP; ProteinTech Group Inc., Chicago, IL, USA), CDK4 (diluted 1:2000; cat. no. 11026-1-AP; ProteinTech Group Inc., Chicago, IL, USA), CDK6 (diluted 1:2000; cat. no. 14052-1-AP; ProteinTech Group Inc., Chicago, IL, USA), cyclin D (diluted 1:2000; cat. no. 26939-1-AP; ProteinTech Group Inc., Chicago, IL, USA), E2F1 (diluted 1:2000; cat. no. 12171-1-AP; ProteinTech Group Inc., Chicago, IL, USA), Foxm1 (diluted 1:1000; cat. no. 5436S; Cell Signaling Technology Inc., USA), Smac (diluted 1:1000; cat. no. 15108S; Cell Signaling Technology, Inc., USA), tubulin (diluted 1:1000; cat. no. 10068-1-AP; ProteinTech Group Inc., Chicago, IL, USA) and β-actin (diluted 1:1000; cat. no. 20536-1-AP; ProteinTech Group Inc., Chicago, IL, USA). The uncropped and unprocessed scans of the most important blots were in the Source Data file.

### Genomic DNA and total RNA isolation from primary cell lines

ESCC PDCs were collected from a 100-mm culture dish, followed by lysing in 600 μL of Buffer RLT Plus additional 14.3 M β-mercaptoethanol, and then for simultaneous purification of genomic DNA and total RNA by the AllPrep DNA/RNA Mini Kit according to the manufacturer’s instructions (80204, Qiagen, Shanghai, China). The yield and quality of DNA and RNA were analyzed by the Agilent 2100 Bioanalyzer.

### Gene expression profiling of primary cell lines

To identify the gene expression profiling of eight ESCC PDCs, RNA-sequencing was performed^53^. Library preparation is the first step. Briefly, after being randomly interrupted, RNAs are converted into a library of cDNA fragments with adaptors attached to both ends of each fragment. Subsequently, the molecules in the library, with amplification, are sequenced and short sequences from both ends (paired-end) are obtained. Randomly interrupt mRNA, cDNA fragments synthesis, RNA library construction, hybrid capture, and sequencing was done at WuXi Next CODE (Shanghai, China). Whole transcriptome sequencing was performed using an Illumina HiSeq X Ten. Expression profiling was quantified as fragments per kilobase of transcript per million mapped reads using featureCounts^54^ after alignment using STAR^55^ with RNA-sequencing data.

### Establishment of ESCC PDX models

Tumor tissues collected at the time of surgery were used to establish the PDX models. Fresh surgical specimens were immediately rinsed in PBS three times and minced into ~1 mm^3^ pieces, which were implanted into the male BALB/c nude mice (*n* = 5 per tumor sample; 4 weeks old, 17–20 g weight; Shanghai, China; license no., SCXK 2007-0005). When the tumors reached ~2 cm at the transplanted position or the mice showed moribund symptoms, nude mice were killed. Xenograft specimens were then collected. These samples with tumorigenic capacity were referred to as “P1.” The “P1” samples were serially passaged in vivo to generate the “P2,” “P3,” and succeeding passages. These were called PDX models. At the same time, the excess tissues were frozen in a cryopreservation liquid containing 49% Dulbecco’s modified Eagle’s medium/nutrient mixture F-12 (Gibco, Shanghai, China), 50% fetal bovine serum (; Gibco, USA), and 1% dimethyl sulfoxide (Sigma-Aldrich Co., USA), and then resuscitations of these cells were performed to test their activities.

### Statistical analysis

Statistical analyses were performed using SPSS (version 18.0, Chicago, IL). A *χ*^2^ test was performed to assess the correlation between the success rate of PDCs and clinicopathological factors. The association between IC50 of CDK4/6 inhibitors and clinical characteristics of ESCC PDC corresponding patients was assessed by multivariate analysis of variance. Data from the experiments were expressed as mean ± SD, based on a minimum of three independent experiments. The comparisons between different groups of compound IC50, cell colony number, tumor volume, and mice weight were performed using Student’s *t* test, and a *p* value of <0.01 and <0.001 was considered significant and very significant. Differences between different groups of gene expression level were compared using Wilcoxon’s test, and a *p* value of <0.05 and <0.01 was considered significant and very significant. To evaluate the differences of IC50 (the sensitivity to compounds) between mutated and non-mutated groups, two-sided *t* test was performed with FDR correction for multiple testing. It was to be noted that the test was conducted when the samples in both mutated and non-mutated groups were not <2. FDR was set to 1 if one of the groups had only 1 sample. The log-rank test and Kaplan–Meier analyses were performed for DFS and OS. *P* < 0.05 was considered statistically significant.

### Reporting summary

Further information on research design is available in the Nature Research Reporting Summary linked to this article.

## Supplementary information

Supplementary Information

Description of Additional Supplementary Files

Supplementary Data 1

Supplementary Data 2

Supplementary Data 3

Supplementary Data 4

Supplementary Data 5

Supplementary Data 6

Supplementary Data 7

Supplementary Data 8

Supplementary Data 9

Supplementary Data 10

Supplementary Data 11

Supplementary Data 12

Supplementary Data 13

Supplementary Data 14

Supplementary Data 15

Supplementary Data 16

Supplementary Data 17

Supplementary Data 18

Supplementary Data 19

Supplementary Data 20

Reporting Summary

## Data Availability

Targeted deep sequencing and RNA-sequencing data have been submitted to NCBI Sequence Read Archive (SRA) (https://www.ncbi.nlm.nih.gov/sra/) with the accession number PRJNA431487. The source data underlying Figs. 3b, 3d, 4a, 4b, 5a, 5c, 6c and Supplementary Fig. 1a, 1b, 5b, 5c, 5 f, 6a, 6b, 6c, 7a, and 7b are provided as a Source Data file. All other data may be found within the main manuscript or supplementary information or available from the authors upon request.

## Code availability

All the related algorithms were described above and additional code used in this study is available upon request.
