# Supplementary material for: Identification of predictors of drug sensitivity using patient-derived models of esophageal squamous cell carcinoma
Source: Nat Commun. 2019 Nov 7;10:5076. doi: 10.1038/s41467-019-12846-7 (PMC6838071; doi:10.1038/s41467-019-12846-7)
Supplement: Supplementary file 1 — Supplementary Information [file 41467_2019_12846_MOESM1_ESM.pdf]

Suppplemental Information

Identification of predictors of drug sensitivity using patient derived models of esophageal squamous cell carcinoma

Su, et al.

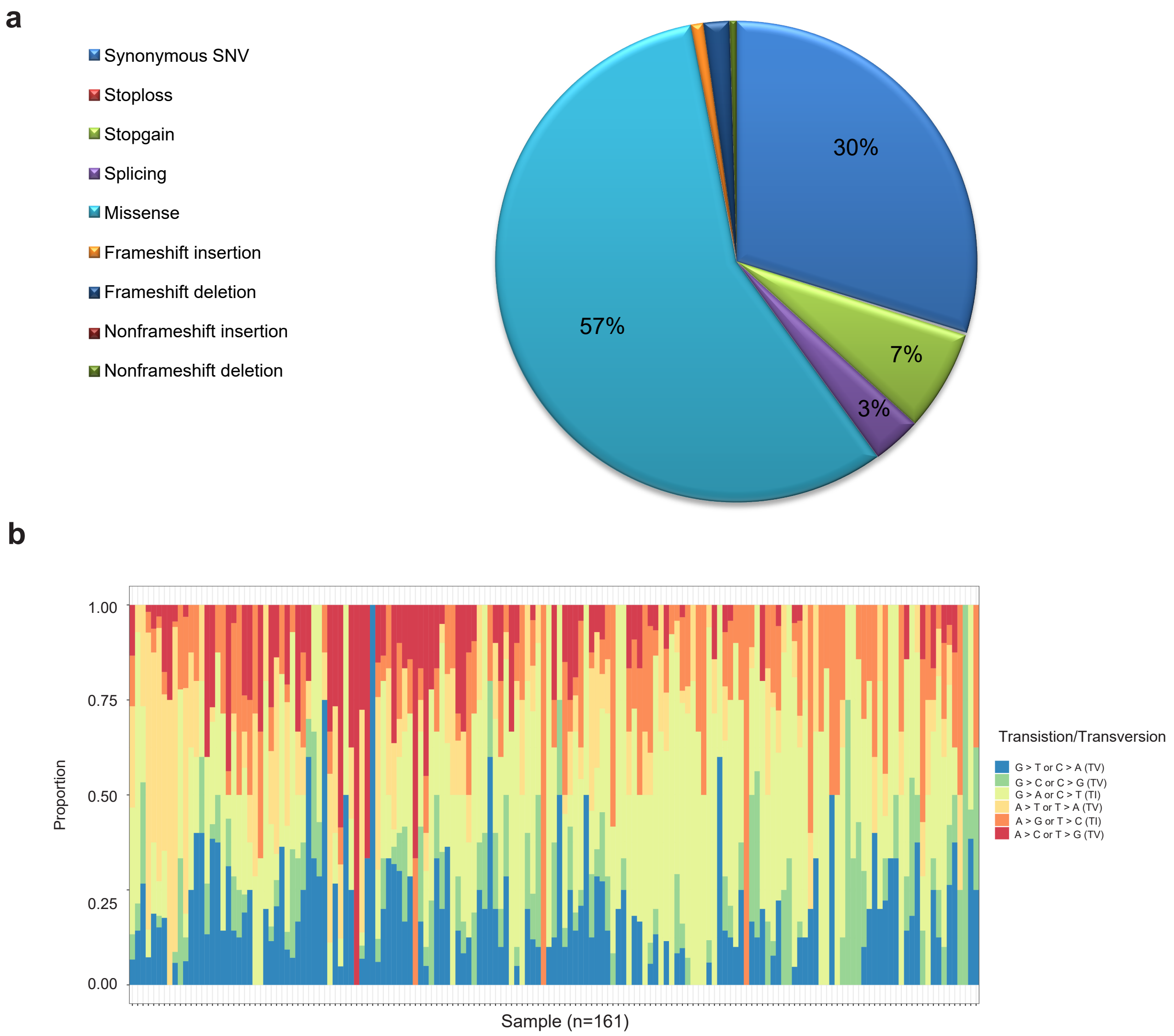

Supplementary Figure 1. Somatic variants distribution and Visualizing transversion and transition substitutions. (a) Somatic variants distribution across genomic functional regions. **(b)** Visualizing transversion and transition substitutions of somatic mutations for 161 FFPE samples.

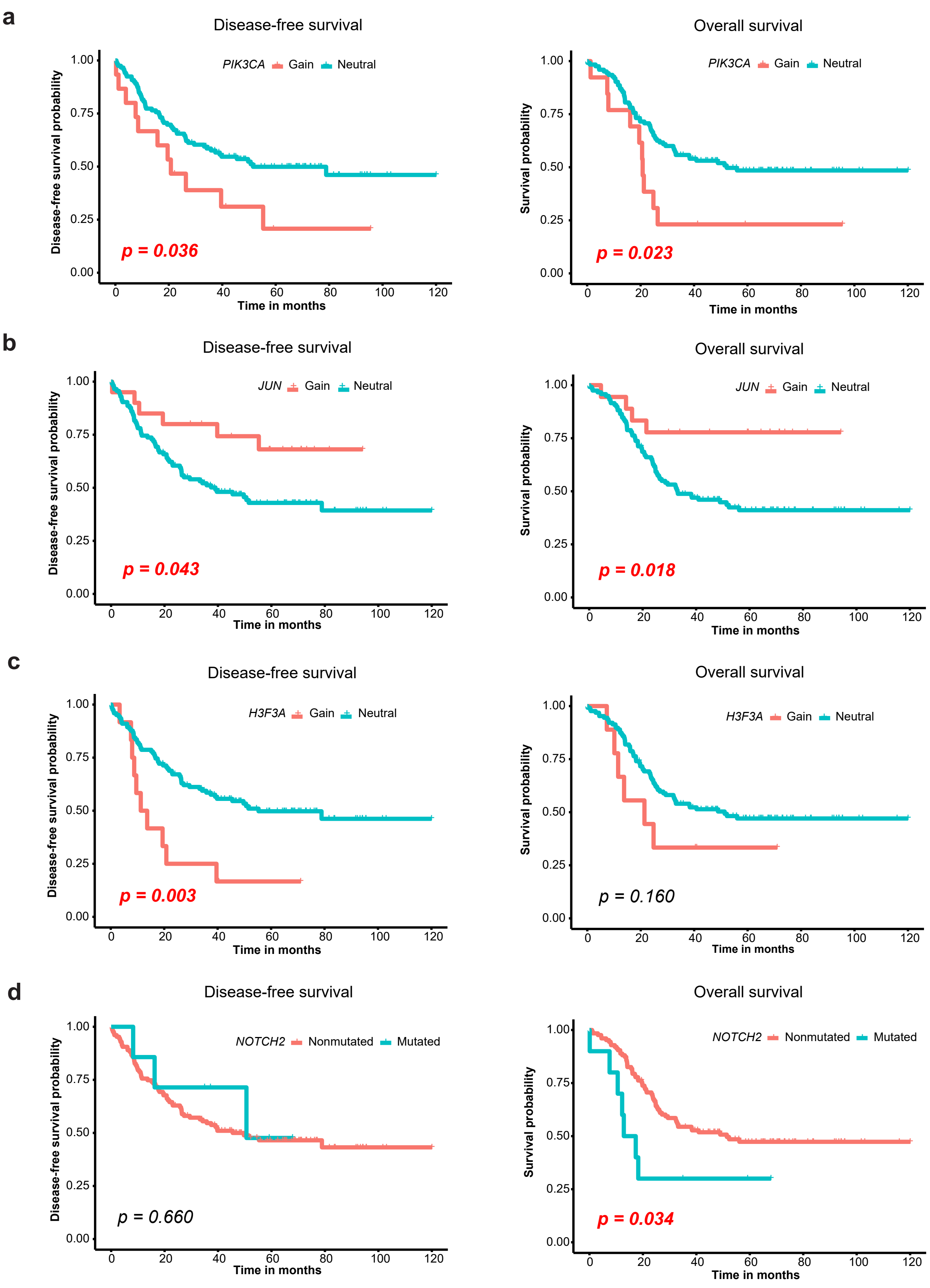
a
Disease-free survival
Disease-free survival probability
PIK3CA Gain Neutral
p = 0.036
Time in months
Overall survival
Survival probability
PIK3CA Gain Neutral
p = 0.023
Time in months
b
Disease-free survival
Disease-free survival probability
JUN Gain Neutral
p = 0.043
Time in months
Overall survival
Survival probability
JUN Gain Neutral
p = 0.018
Time in months
c
Disease-free survival
Disease-free survival probability
H3F3A Gain Neutral
p = 0.003
Time in months
Overall survival
Survival probability
H3F3A Gain Neutral
p = 0.160
Time in months
d
Disease-free survival
Disease-free survival probability
NOTCH2 Nonmutated Mutated
p = 0.660
Time in months
Overall survival
Survival probability
NOTCH2 Nonmutated Mutated
p = 0.034
Time in months

e

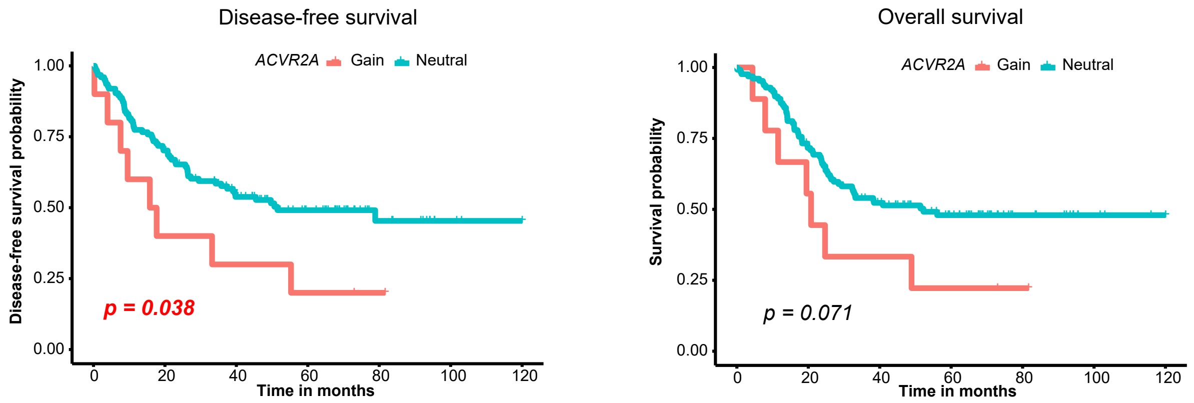

f

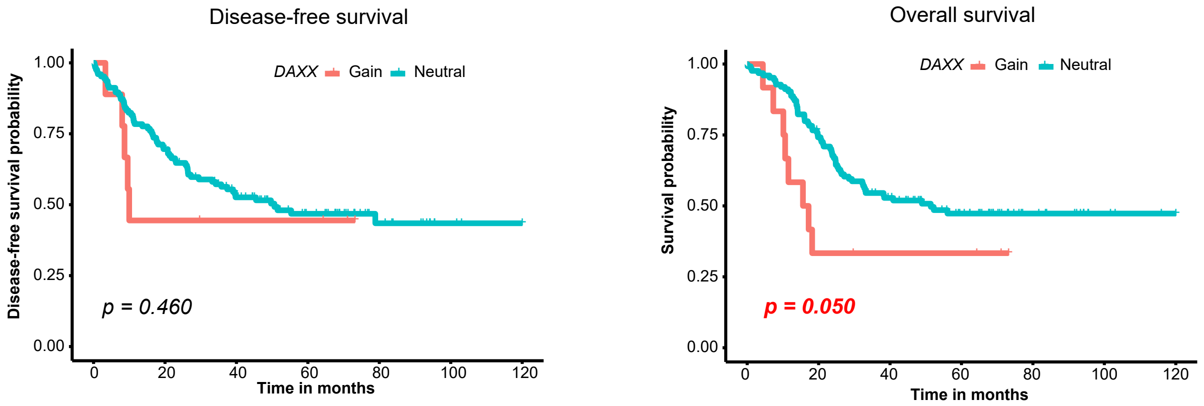

Supplementary Figure 2. Cancer gene mutations and prognosis. Kaplan-Meier statistical analyses were performed on progression-free survival (PFS) and overall survival (OS) data of ESCC patients with *PIK3CA* gain and neutral mutations **(a)**, *JUN* gain and neutral mutations **(b)**, *H3F3A* gain and neutral mutations **(c)** and *NOTCH2* mutation and non-mutation **(d)**, *ACVR2A* gain and neutral mutations **(e)** and *DAXX* gain and neutral mutations **(f)**. The log-rank test and Kaplan-Meier analyses were performed for DFS and OS. $P < 0.05$ was considered statistically significant.

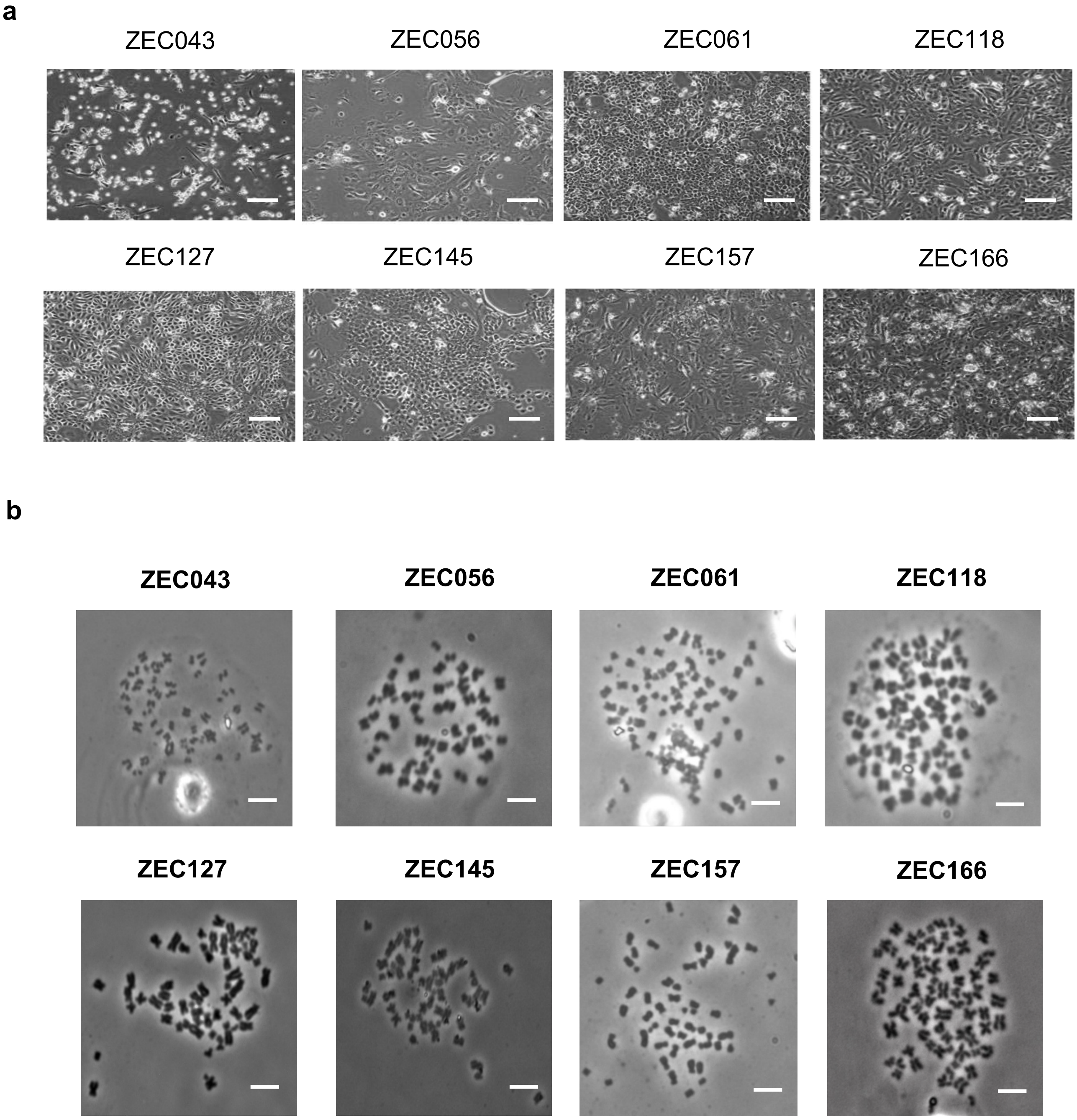
a
ZEC043
ZEC056
ZEC061
ZEC118
ZEC127
ZEC145
ZEC157
ZEC166
b
ZEC043
ZEC056
ZEC061
ZEC118
ZEC127
ZEC145
ZEC157
ZEC166

c

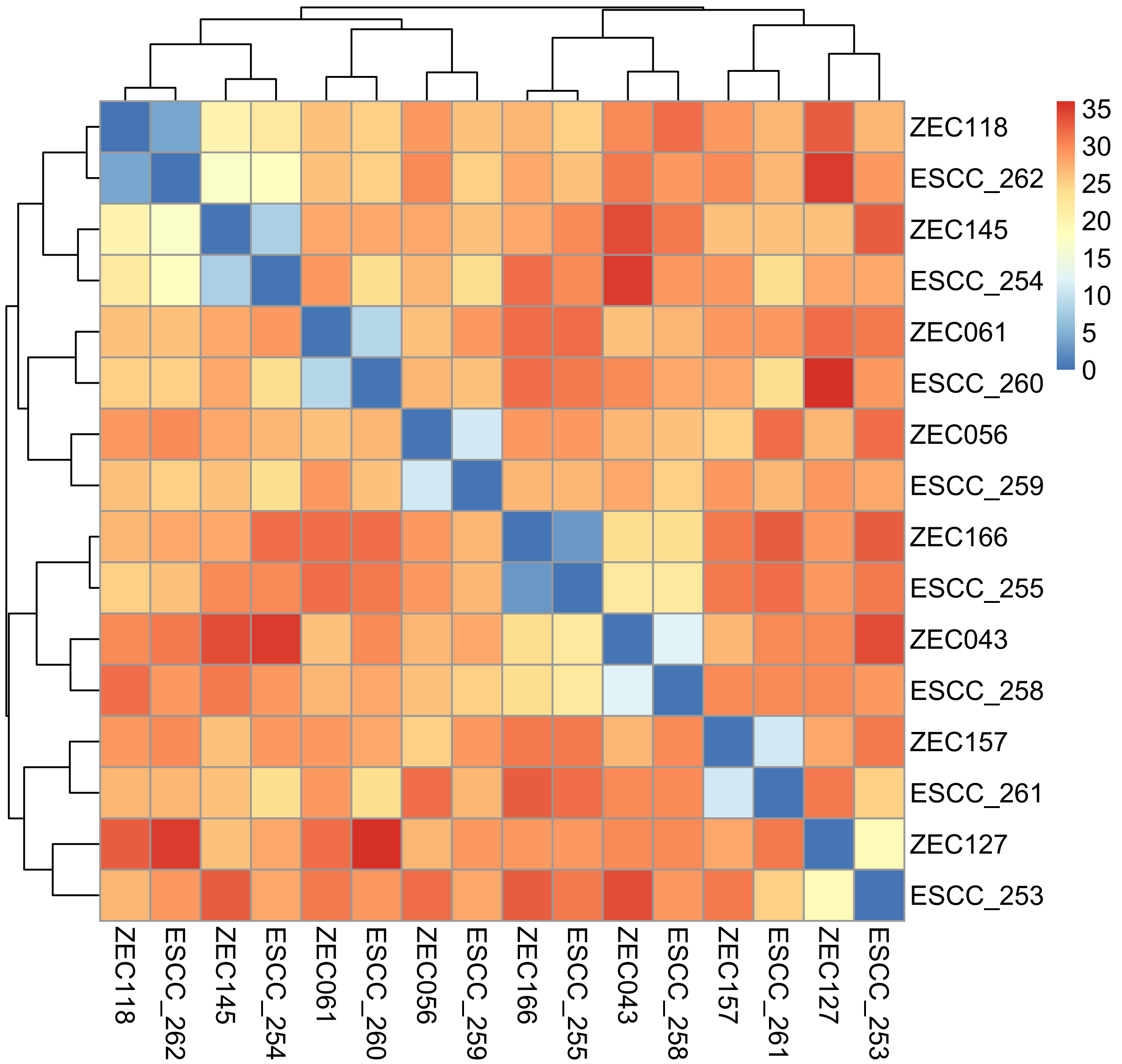

Supplementary Figure 3. The validation of ESCC patient-derived cells. (a) Cell morphology are distinct across eight patient-derived cells. Scale bar 50 μm. **(b)** Imaging results from karyotyping are distinct across eight patient-derived cells. Scale bar 5 μm. **(c)** The consistency of SNPs in eight ESCC PDCs and corresponding tissues. The clustering analysis depended on the SNP differences in the samples. The colors from blue to red represent the number of different SNPs from 0 to 35.

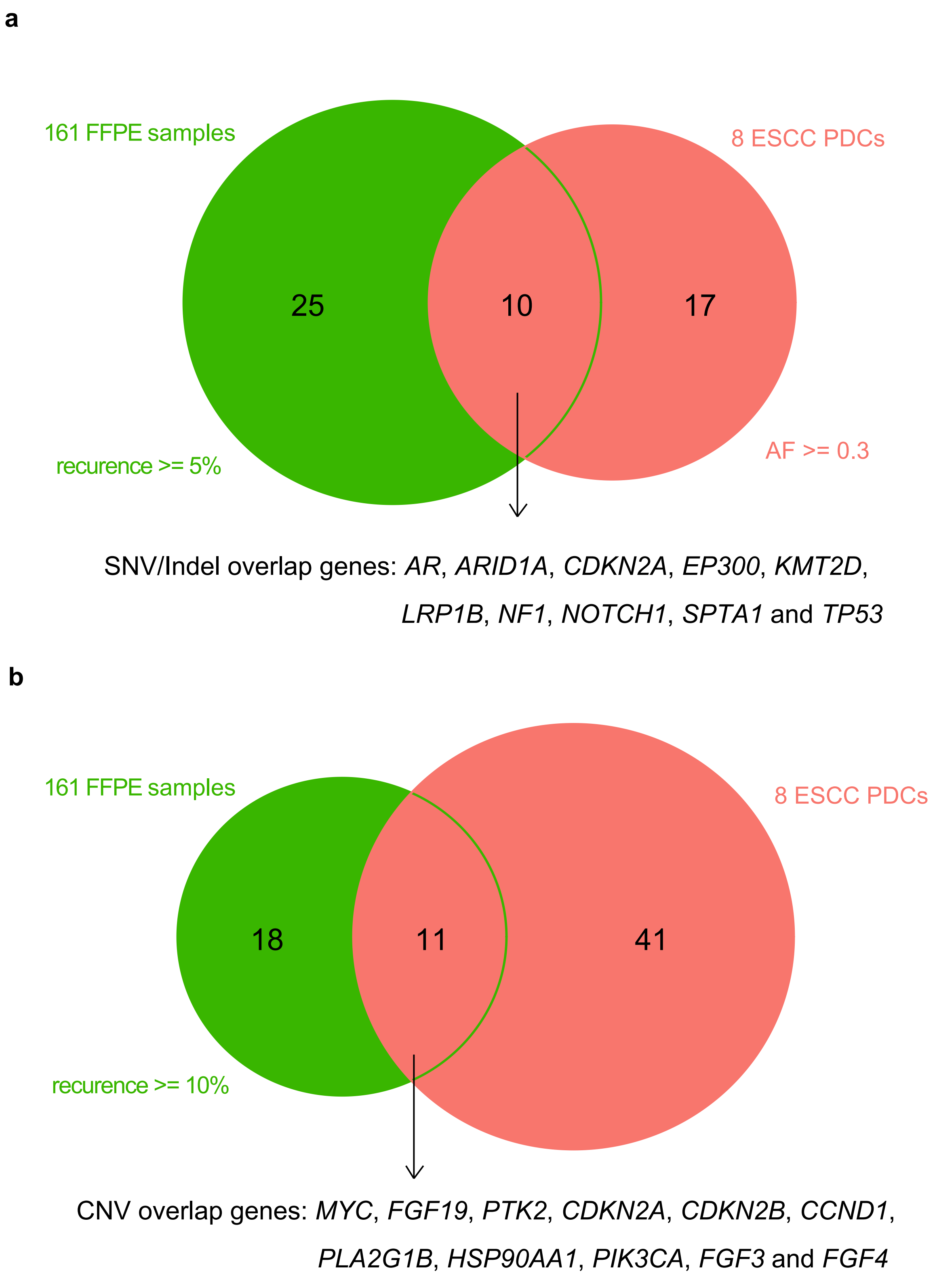

Supplementary Figure 4. The SNV/indel and CNV overlapping genes between the eight PDCs and the 161 ESCC patients. (a) The SNV/indel overlapping genes between the eight PDCs (Allele frequency >= 30%) and the 161 ESCC patients (recurrence >= 5%) included *AR*, *ARID1A*, *CDKN2A*, *EP300*, *KMT2D*, *LRP1B*, *NF1*, *NOTCH1*, *SPTA1*, and *TP53*. **(b)** The CNV overlapping genes between the eight PDCs and the 161 ESCC patients (recurrence >= 10%) contained *MYC*, *FGF19*, *PTK2*, *CDKN2A*, *CDKN2B*, *CCND1*, *PLA2G1B*, *HSP90AA1*, *PIK3CA*, *FGF3*, and *FGF4*.

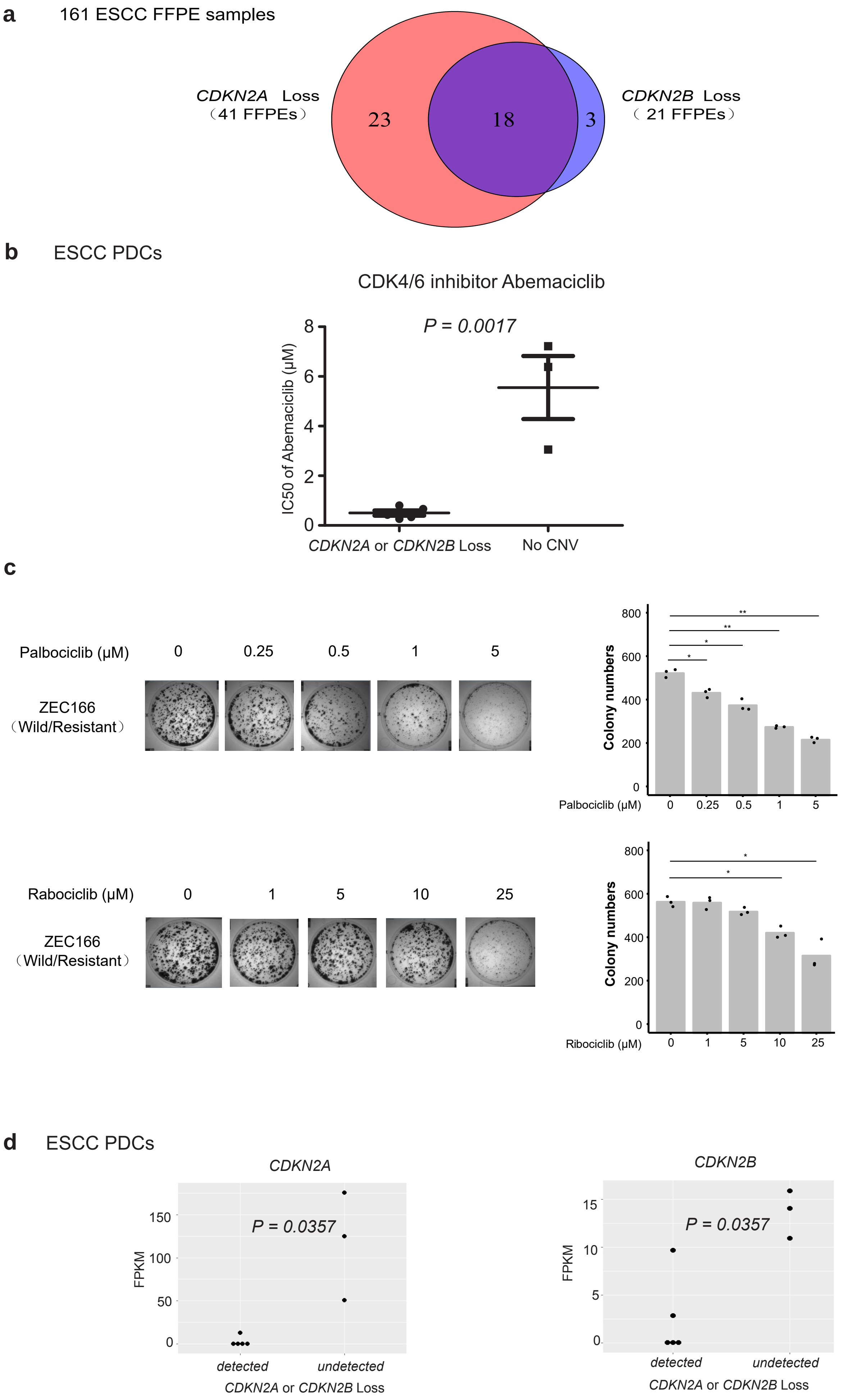
a
161 ESCC FFPE samples
CDKN2A Loss
(41 FFPEs)
CDKN2B Loss
(21 FFPEs)
23
18
3
b
ESCC PDCs
CDK4/6 inhibitor Abemaciclib
P = 0.0017
IC50 of Abemaciclib (μM)
8
6
4
2
0
CDKN2A or CDKN2B Loss
No CNV
c
Palbociclib (μM)
0
0.25
0.5
1
5
ZEC166
(Wild/Resistant)
Colony numbers
800
600
400
200
0
Palbociclib (μM)
**
**
*
*
Rabociclib (μM)
0
1
5
10
25
ZEC166
(Wild/Resistant)
Colony numbers
Ribociclib (μM)
*
*
d
ESCC PDCs
CDKN2A
P = 0.0357
FPKM
150
100
50
0
detected
undetected
CDKN2A or CDKN2B Loss
CDKN2B
P = 0.0357
FPKM
15
10
5
0
detected
undetected
CDKN2A or CDKN2B Loss

e Commerical cell lines

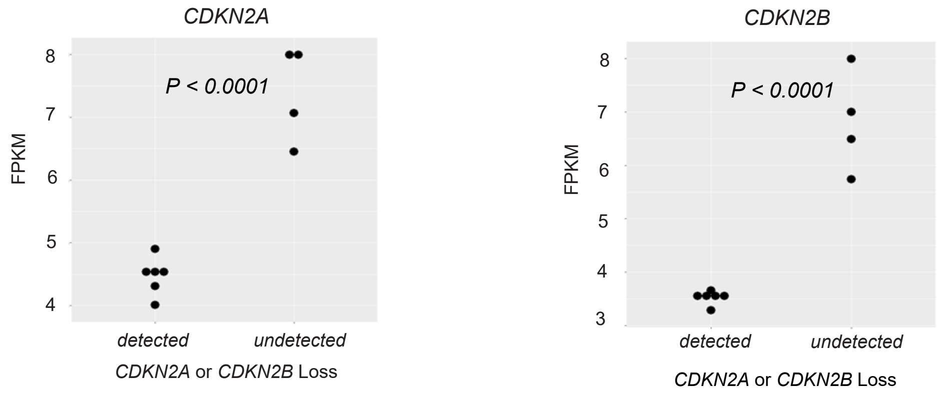

f

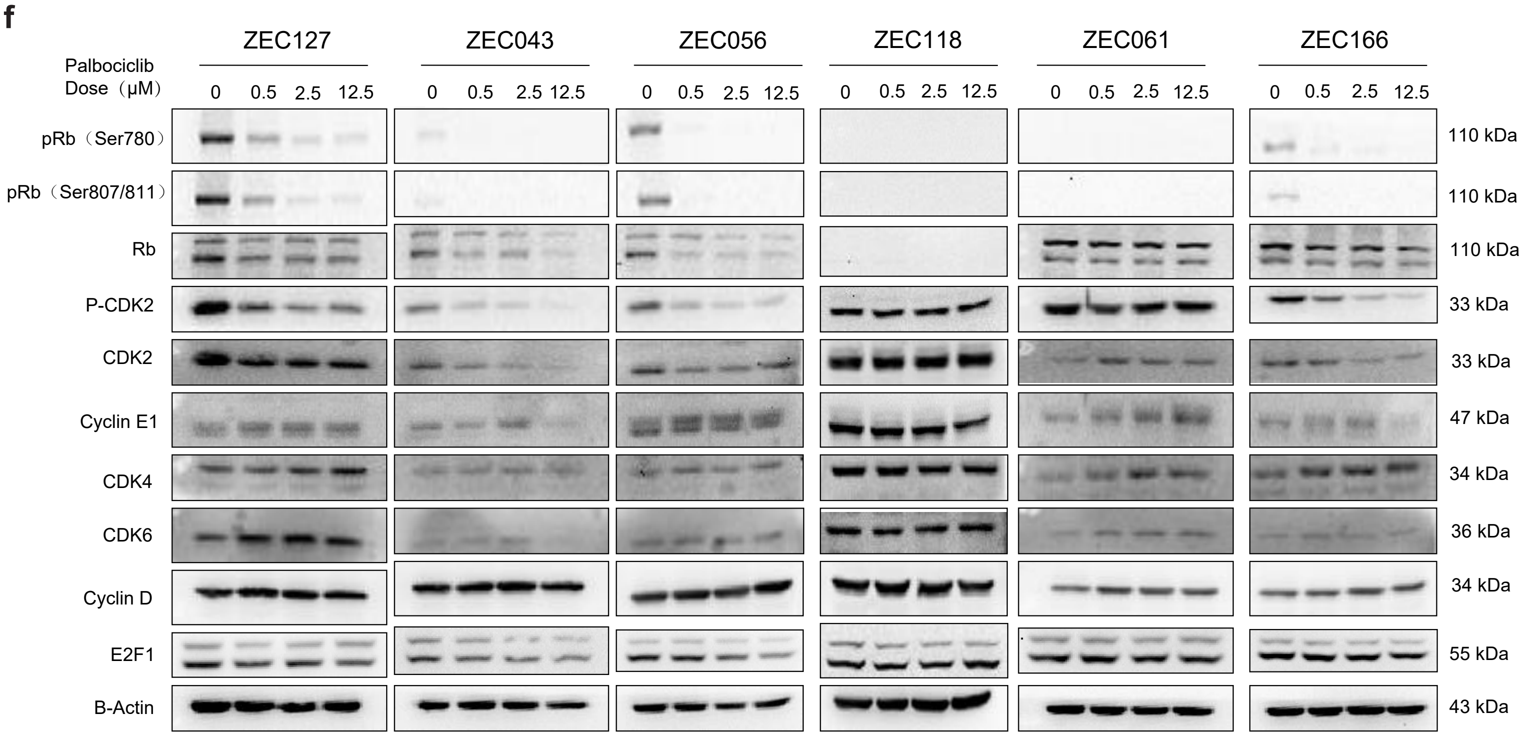

Supplementary Figure 5. The validation of ESCC patient-derived cells. (a) The relationship of 44 ESCC FFPE samples harboring *CDKN2A* or *CDKN2B* loss from the 161 ESCC FFPE samples is shown. **(b)** Fifty percent growth inhibitory concentrations (IC50s) of abemaciclib in ESCC PDCs with *CDKN2A* or *CDKN2B* loss or with no CNV of *CDKN2A* and *CDKN2B*. Four experiments were averaged, error bars correspond to the standard deviation of Abemaciclib IC50s. The comparisons between different groups of compound IC50s were performed using Student's t test, and a p value of < 0.01 was considered significant. **(c)** ZEC166 (*CDKN2A/2B* no CNV) were used to evaluate the sensitivity of CDK4/6 inhibitors (palbociclib and ribociclib) using colony formation assays. Three experiments were averaged, dots correspond to colony numbers. The colony number differences between different dose groups of inhibitors were compared using a multiple t test and * $p < 0.01$ and ** $p < 0.001$. **(d)** and **(e)** The correlation analysis between somatic CNVs of *CDKN2A* or *CDKN2B* and their relative expressions detected through RNA sequencing in 8 ESCC PDCs and 10 ESCC commercial cell lines. **(f)** Western blot analysis of lysates from ESCC PDCs (ZEC043, ZEC056, ZEC127, ZEC118, ZEC061, and ZEC166) blotted with the indicated antibodies. The concentrations of palbociclib were, respectively, 0 μM, 0.5 μM, 2.5 μM, and 12.5 μM. The uncropped and unprocessed scans of blots data are provided in a Source Data file.

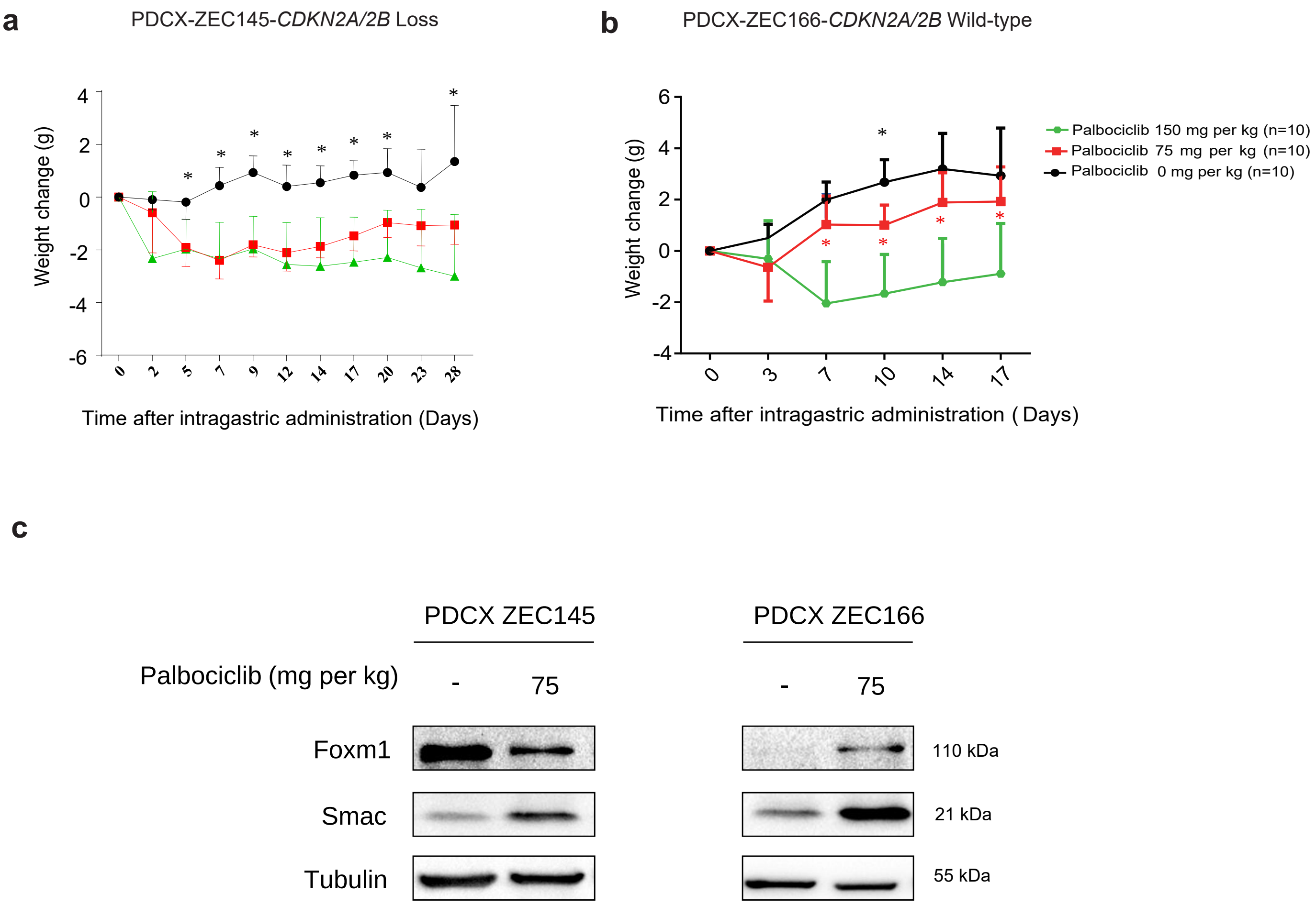

Supplementary Figure 6. Change weight of mice following treatment with palbociclib in PDC xenografts and the expression levels of potential markers of CDK4/6 inhibitor activity. (a) and **(b)** Change in weight following treatment with palbociclib in three doses over 28 days in ESCC PDCX (ZEC145) and PDCX (ZEC166) (mean ± s.e.m., n = 10). *: $p <$ 0.01, multiple t test, vehicle compared with Palbociclib 75 mg per kg. Error bars correspond to standard error of the mean of tumor volume. *: $p < 0.01$, multiple t test, Palbociclib 75 mg per kg compared with Palbociclib 150 mg per kg. **(C)** Western blot analysis of lysates from tumor tissue samples of PDC xenografts (PDCX ZEC043 and PDCX ZEC166) blotted with the indicated antibodies. The *in vivo* concentrations of palbociclib were, respectively, 0 mg per kg and 75 mg per kg. The uncropped and unprocessed scans of blots data are provided in a Source Data file.

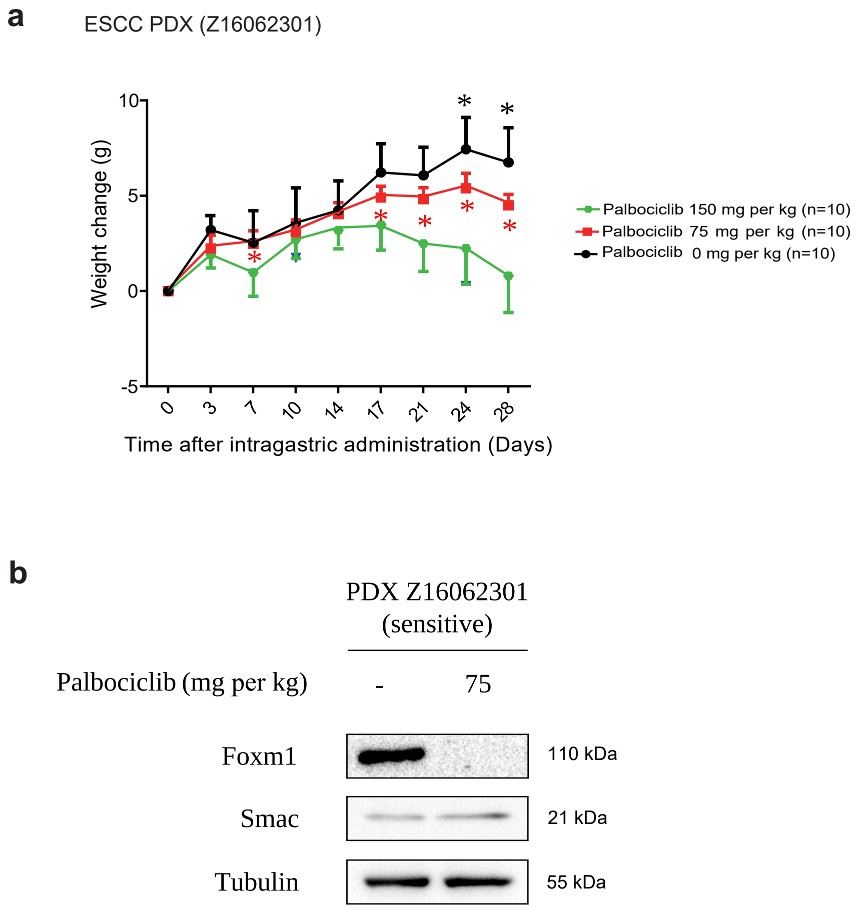

Supplementary Figure 7. Change weight of mice following treatment ith palbociclib in ESCC xenografts and the expression levels of potential markers of CDK4/6 inhibitor activity. (a) Change in weight following treatment with palbociclib at three doses over 28 days in ESCC PDX-Z16062301 (mean ± s.e.m., n = 10). *: *p* < 0.01, multiple t test, vehicle compared with Palbociclib 75 mg per kg. Error bars correspond to standard error of the mean of tumor volume. *: *p* < 0.01, *Student's t test*, Palbociclib 75 mg per kg compared with Palbociclib 150 mg per kg. **(b)** Western blot analysis of lysates from tumor tissue samples of an ESCC PDX model (PDX-Z16062301) blotted with the indicated antibodies. The *in vivo* concentrations of palbociclib were, respectively, 0 mg per kg and 75 mg per kg.

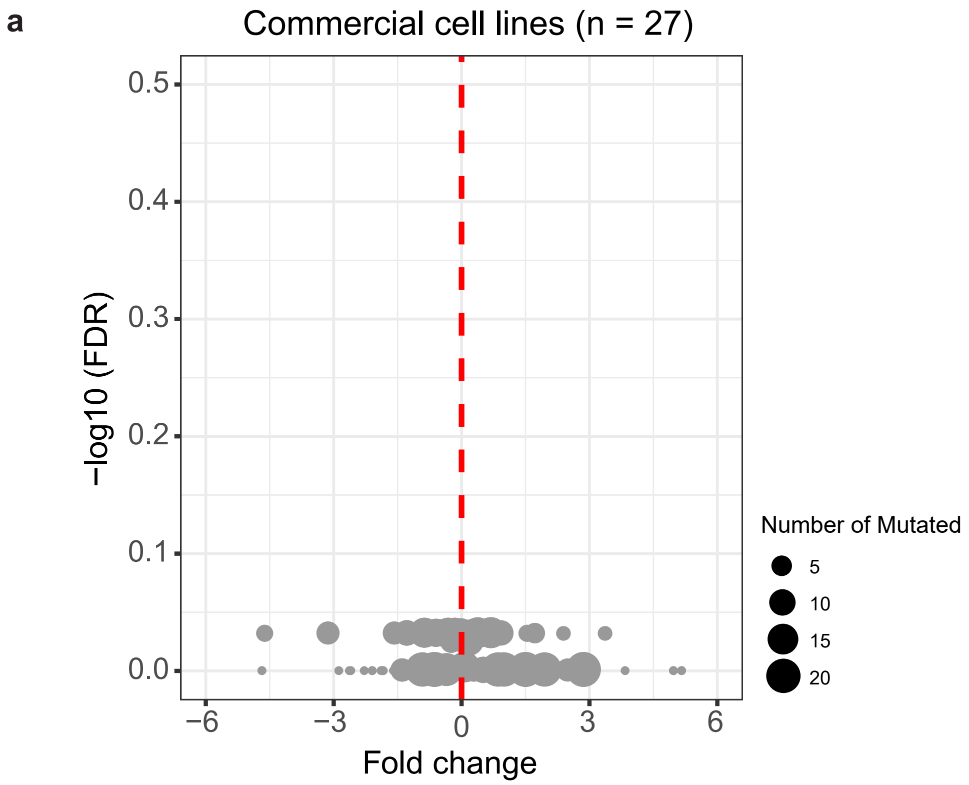

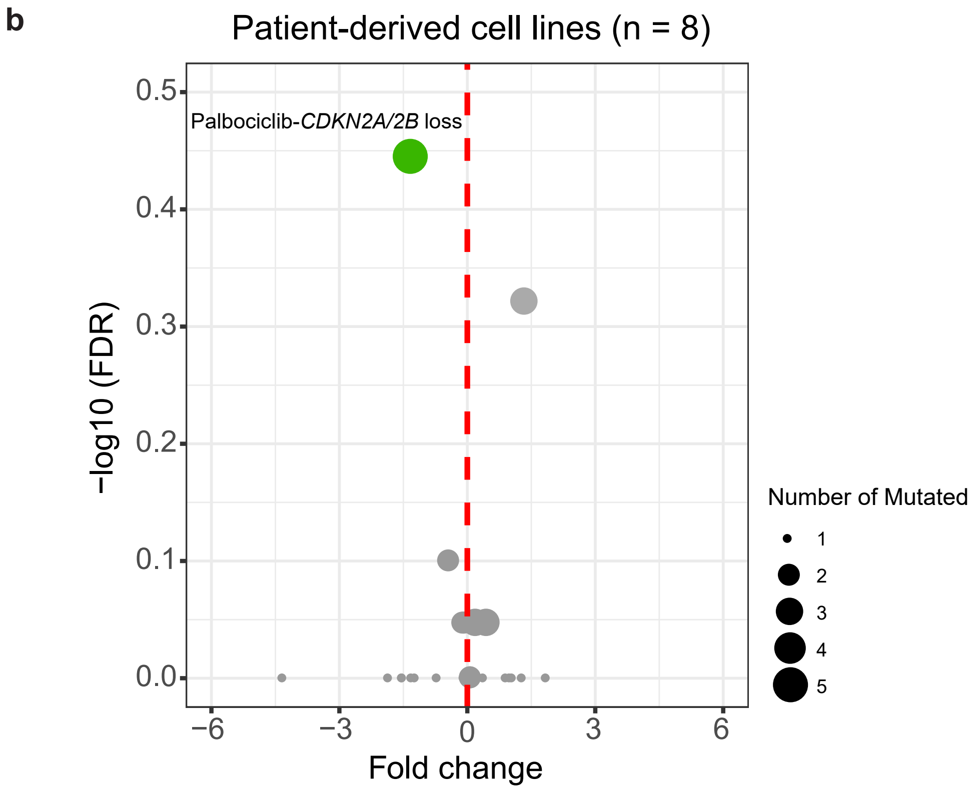

Supplementary Figure 8. To identify the biomarkers of drug sensitivity, we analyzed the overlapping 14 compounds on 27 commercialized ESCC cell lines from the CCLE project and 8 ESCC PDCs from our platform. Each circle represents a single drug-gene interaction, and the size is proportional to the number of mutant cell lines screened . An unpaired t-test was performed for each drug-gene interaction. To evaluate the differences of IC50 (the sensitivity to compound) between mutated and non-mutated groups, two-sided t-test was performed with FDR correction for multiple testing. It was to be noted that test was conducted when the samples in both mutated and non-mutated groups were not less than two. FDR was set to 1 if one of the groups had only 1 sample. The drug-gene associations of 27 commercialized ESCC cell lines **(a)** and 8 ESCC PDCs **(b)** were separately displayed. The top drug-gene mutation association Palbociclib-*CDKN2A/2B* loss in ESCC PDCs sorted by FDR were colored by blue.
