# Supplementary material for: Identification of predictors of drug sensitivity using patient-derived models of esophageal squamous cell carcinoma
Source: Nat Commun. 2019 Nov 7;10:5076. doi: 10.1038/s41467-019-12846-7 (PMC6838071; doi:10.1038/s41467-019-12846-7)
Supplement: Supplementary file 2 — Description of Additional Supplementary Files [file 41467_2019_12846_MOESM2_ESM.pdf]

Description of Additional Supplementary Files

File Name: Supplementary Data 1
Description: Clinical information of 161 patients with esophageal squamous cell carcinoma (ESCC).

File Name: Supplementary Data 2
Description: Gene list of deep sequencing.

File Name: Supplementary Data 3
Description: Quality control of deep sequence data.

File Name: Supplementary Data 4
Description: Single nucleotide variants (SNVs) and Indels results of 161 ESCC samples.

File Name: Supplementary Data 5
Description: Copy number variations (CNVs) of 161 ESCC samples.

File Name: Supplementary Data 6
Description: Clinical information of 123 patients with ESCC and associations between the success rate of patient-derived cancer cell line (PDC) establishment and these clinical characteristics.

File Name: Supplementary Data 7
Description: Karyotype results of eight ESCC PDCs.

File Name: Supplementary Data 8
Description: Short tandem repeat results of eight ESCC PDCs.

File Name: Supplementary Data 9
Description: SNVs and Indels results of eight ESCC PDCs.

File Name: Supplementary Data 10
Description: CNVs of eight ESCC PDCs.

File Name: Supplementary Data 11
Description: Potential drug-genotype associations in eight ESCC PDCs.

File Name: Supplementary Data 12
Description: Half-maximal inhibitory concentrations (IC50s) of 46 compounds on eight ESCC PDCs.

File Name: Supplementary Data 13

Description: Analysis of drug-gene associations of eight ESCC PDCs in 46 compounds.

File Name: Supplementary Data 14
Description: IC50s of CDK4/6 inhibitors palbocicilib, ribocicilib, and abemaciclib on eight ESCC PDCs in the validation.

File Name: Supplementary Data 15
Description: Clinical characteristics of eight PDC corresponding patients and associations between clinical characteristics of eight PDC corresponding patients and sensitivities of palbociclib and ribociclib.

File Name: Supplementary Data 16
Description: Mutational profiles of 10 commercial cell lines from CCLE project.

File Name: Supplementary Data 17
Description: Difference analysis of mRNA level between mutated PDCs and non-mutated PDCs of cell cycle regulation genes.

File Name: Supplementary Data 18
Description: Clinical information of an ESCC patient whose tumor tissue was used for patient-derived xenografts (PDXs).

File Name: Supplementary Data 19
Description: Mutational profile of ESCC PDX model.

File Name: Supplementary Data 20
Description: IHC results of p15, p16, and Ki67 in FFPE tumor tissues from PDXs treated with different dosages of palbociclib.
