# Supplementary material for: Identification of predictors of drug sensitivity using patient-derived models of esophageal squamous cell carcinoma
Source: Nat Commun. 2019 Nov 7;10:5076. doi: 10.1038/s41467-019-12846-7 (PMC6838071; doi:10.1038/s41467-019-12846-7)
Supplement: Supplementary file 23 — Reporting Summary [file 41467_2019_12846_MOESM23_ESM.pdf]

nature research

Corresponding author(s): Prof. Dan Su and Prof. Weimin Mao

Last updated by author(s): Sep 5, 2019

Reporting Summary

Nature Research wishes to improve the reproducibility of the work that we publish. This form provides structure for consistency and transparency in reporting. For further information on Nature Research policies, see Authors & Referees and the Editorial Policy Checklist .

Statistics

For all statistical analyses, confirm that the following items are present in the figure legend, table legend, main text, or Methods section.

n/a	Confirmed	
[ ]	[x]	The exact sample size (*n*) for each experimental group/condition, given as a discrete number and unit of measurement
[ ]	[x]	A statement on whether measurements were taken from distinct samples or whether the same sample was measured repeatedly
[ ]	[x]	The statistical test(s) used AND whether they are one- or two-sided<br>*Only common tests should be described solely by name; describe more complex techniques in the Methods section.*
[x]	[ ]	A description of all covariates tested
[ ]	[x]	A description of any assumptions or corrections, such as tests of normality and adjustment for multiple comparisons
[ ]	[x]	A full description of the statistical parameters including central tendency (e.g. means) or other basic estimates (e.g. regression coefficient) AND variation (e.g. standard deviation) or associated estimates of uncertainty (e.g. confidence intervals)
[ ]	[x]	For null hypothesis testing, the test statistic (e.g. *F*, *t*, *r*) with confidence intervals, effect sizes, degrees of freedom and *P* value noted<br>*Give P values as exact values whenever suitable.*
[x]	[ ]	For Bayesian analysis, information on the choice of priors and Markov chain Monte Carlo settings
[x]	[ ]	For hierarchical and complex designs, identification of the appropriate level for tests and full reporting of outcomes
[x]	[ ]	Estimates of effect sizes (e.g. Cohen's *d*, Pearson's *r*), indicating how they were calculated

Our web collection on statistics for biologists contains articles on many of the points above.

Software and code

Policy information about availability of computer code

Data collection	FastQC software was used to evaluate the quality of sequencing data (http://www.bioinformatics.bbsrc.ac.uk/projects/fastqc/). Sequence reads from genomic DNA were mapped to human genome (hg19) reference using BWA-MEM, and bam files were further processed by Picard (http://broadinstitute.github.io/picard/) to sort sequences and remove duplicated reads.
Data analysis	Somatic single nucleotide variants (SNVs) were called by Mutect, and Indels (less than 50 bp) were identified by Pindel and VarScan. ANNOVAR software were utilized to facilitate variants annotation. Statistical analyses were performed using SPSS (version 18.0, Chicago, IL)

For manuscripts utilizing custom algorithms or software that are central to the research but not yet described in published literature, software must be made available to editors/reviewers. We strongly encourage code deposition in a community repository (e.g. GitHub). See the Nature Research guidelines for submitting code & software for further information.

Data

Policy information about availability of data

All manuscripts must include a data availability statement. This statement should provide the following information, where applicable:

- Accession codes, unique identifiers, or web links for publicly available datasets
- A list of figures that have associated raw data
- A description of any restrictions on data availability

Targeted deep sequencing and RNA-seq data supporting the findings of this study have been submitted to NCBI Sequence Read Archive (SRA) (https://submit.ncbi.nlm.nih.gov/subs/sra) with the permanent accession number SUB3428867.

Field-specific reporting

Please select the one below that is the best fit for your research. If you are not sure, read the appropriate sections before making your selection.

☒ Life sciences ☐ Behavioural & social sciences ☐ Ecological, evolutionary & environmental sciences

For a reference copy of the document with all sections, see nature.com/documents/nr-reporting-summary-flat.pdf

Life sciences study design

All studies must disclose on these points even when the disclosure is negative.

Sample size	Because this study is not a clinical trial of drugs or a comparative study of groups, there is no sample-size calculation in this study. The fresh tumor tissues from 123 ESCC patients which were collected in this study were used to establish patient-derived primary cell lines (PDCs). An independent cohort including 161 ESCC cancer tissues were used to profile the actionable mutations in ESCC. These samples were collected as best we could before the detection deadline.
Data exclusions	There is no data exclusion in the analyses of this study.
Replication	For the cell viability assay with compounds in vitro, mean IC50 values and SDs were calculated from two independent biological replications. In colony formation assay, the inhibitions of CDK4/6 inhibitors on esophageal squamous cell carcinoma patient-derived primary cell lines were calculated from three independent experiments.
Randomization	161 formalin-fixed paraffin-embedded (FFPE) tumor tissues and matched blood samples, as well as 123 fresh tumor tissues were allocated randomizedly by the tissue bank of Zhejiang Cancer Hospital.
Blinding	Because this study did not involve clinical sample grouping, we were not blinded to group allocation during data collection and analyses.

Reporting for specific materials, systems and methods

We require information from authors about some types of materials, experimental systems and methods used in many studies. Here, indicate whether each material, system or method listed is relevant to your study. If you are not sure if a list item applies to your research, read the appropriate section before selecting a response.

Materials & experimental systems

n/a	Involved in the study	
☐	☒	Antibodies
☒	☐	Eukaryotic cell lines
☒	☐	Palaeontology
☐	☒	Animals and other organisms
☐	☒	Human research participants
☐	☒	Clinical data

Methods

n/a	Involved in the study	
☒	☐	ChIP-seq
☒	☐	Flow cytometry
☒	☐	MRI-based neuroimaging

Antibodies

Antibodies used	The following antibodies were used: pRb (Ser780) (1:1,000, Cell Signaling Technology, Inc., USA), pRb (Sr807/811) (1:1,000, Cell Signaling Technology, Inc., USA), Rb (1:800, Cell Signaling Technology, Inc., USA), P-CDK2 (1:1,000, Cell Signaling Technology, Inc., USA), CDK2 (1:1,000, Cell Signaling Technology, Inc., USA), CyclinE (1:2,000, ProteinTech Group, Inc., Chicago, IL, USA), CDK4 (1:2,000, ProteinTech Group, Inc., Chicago, IL, USA), CDK6 (1:2,000, ProteinTech Group, Inc., Chicago, IL, USA), CyclinD (1:2,000, ProteinTech Group, Inc., Chicago, IL, USA), E2F (1:2,000, ProteinTech Group, Inc., Chicago, IL, USA), Foxm1 (diluted 1:1,000; cat. no. 5436S; Cell Signaling Technology, Inc., USA), Smac (diluted 1:1,000; cat. no. 15108S; Cell Signaling Technology, Inc., USA), Tubulin (diluted 1:1,000; cat. no. 10068-1-AP; ProteinTech Group, Inc., Chicago, IL, USA), and beta-Actin (1:1,000, ProteinTech Group, Inc., Chicago, IL, USA).
Validation	pRb (Ser780)-COS and Jurkat cells,<br>pRb (Sr807/811)- MCF7 cells and WI-38 cells,<br>Rb- WI-38 cells,<br>P-CDK2 -HeLa cells,<br>CDK2 -HeLa, NIH/3T3, C6 and COS cells,<br>CyclinE - HeLa cells, Jurkat cells and MCF7 cells,<br>CDK4-HepG2 cells and Hela cells,<br>CDK6-C2C12 and Jurkat cells ,<br>CyclinD-A549 and Hela cells,<br>E2F-A431 and Hela cells,

	Foxm1-Hela cells,<br>Smac-Jurkat cells<br>Tubulin-HEK-293 cells<br>beta-Actin –A549 and HEK-293 cells.

Animals and other organisms

Policy information about studies involving animals; ARRIVE guidelines recommended for reporting animal research

Laboratory animals	The male BALB/c nude mice, which were 4 weeks old with a weight of 17–20 g, were used in this study.
Wild animals	This study did not involve wild animals.
Field-collected samples	After 28 days or when the tumor volume had reached 1500 mm3, the mice were killed. Samples were collected in the Biological Laboratory of Animal Center, Zhejiang University of Traditional Chinese Medicine, at room temperature of 20-25 ℃. After a group of animals were executed, they were photographed immediately.
Ethics oversight	Animal experiments were approved by the Institutional Animal Care and Use Committee of Zhejiang Chinese Medicine University (Hangzhou, Zhejiang, China).

Note that full information on the approval of the study protocol must also be provided in the manuscript.

Human research participants

Policy information about studies involving human research participants

Population characteristics	Among 161 patients with ESCC , 87.0% (140 patients) were male respectively, 79.5% (128 patients) were equal to or less than 65 years old, 73.9% (119 patients) had BMI from 18.5 to 25, 77.0% (124 patients) had smoking status, 72.7% (117 patients) had alcohol intake, 29.8% (48 patients) had family history, 59.6% (96 patients) of tumors were from the middle esophagus, 73.3% (118 patients) were moderately differentiated. Based on the seventh edition of the American Joint Committee on Cancer (AJCC) staging system for esophageal cancer (22), 1 (0.6%) patient was stage I, 4 (2.5%) patients were stage II, 91 (56.5%) patients were stage IIIa, 47 (29.2%) patients were stage IIIb, and 18 (11.2%) patients were stage IIIc.
Recruitment	All samples were obtained from the tissue bank of Zhejiang Cancer Hospital
Ethics oversight	All samples were collected with written inform consents and this study was approved by the Institutional Review Board in Zhejiang Cancer Hospital.

Note that full information on the approval of the study protocol must also be provided in the manuscript.

Clinical data

Policy information about clinical studies

All manuscripts should comply with the ICMJEguidelines for publication of clinical research and a completedCONSORT checklist must be included with all submissions.

Clinical trial registration	This study is not a clinical trial but involve in the association study between gene variations and clinical factors in 161 ESCC patients. No a clinical trial registration.
Study protocol	Because this study is not a clinical trial, there is not a trial protocol.
Data collection	To investigate the associations of mutations with clinical outcomes, clinical information and ten years follow-up of 161 ESCC patients were collected from August 2008 to July 2018 in Zhejiang Cancer Hospital. To establish patient-derived primary cells, an independent cohort including 123 ESCC patients were enrolled and their clinical information were collected from October 2014 to August 2017.
Outcomes	The disease-free survival (DFS) of patients was measured as the duration from surgery to the time of tumor recurrence or last follow-up evaluation. The overall survival (OS) was defined as the duration from surgery to the time of death or last follow-up evaluation. We analyzed the associations of gene variations with DFS and OS in 161 ESCC patients.
